# Global trends, projections, and inequalities in overweight and obesity among children and adolescents from 1990 to 2040: a modelling study

**DOI:** 10.3389/fendo.2026.1855271

**Published:** 2026-09-22

**Authors:** Xuelong Su, Jinli Liu, Mingwang Shen

**Affiliations:** 1 Key Laboratory of Shaanxi Province for Craniofacial Precision Medicine Research, College of Stomatology, Xi’an Jiaotong University, Xi’an, China; 2 School of Public Health, Xi’an Jiaotong University Health Science Center, Xi’an, Shaanxi, China; 3 Department of Endocrinology, The First Affiliated Hospital of Xi’an Jiaotong University, Xi’an Jiaotong University, Xi’an, Shaanxi, China

**Keywords:** obesity, overweight, child, adolescent, projection, health inequalities, global health

## Abstract

**Background:**

Evidence remains limited regarding how childhood and adolescent overweight and obesity have evolved across countries at different levels of economic development and how these patterns may change in the future. We therefore characterised global trends from 1990 to 2022, generated projections to 2040, and examined changes in absolute and relative GDP-related inequalities.

**Methods:**

We analysed NCD-RisC prevalence estimates for children and adolescents aged 5–19 years in 200 countries and territories. Joinpoint regression was used to assess temporal trends and estimate average annual percentage changes (AAPCs) in prevalence. Bayesian age-period-cohort models were used to project prevalence to 2040. Population-weighted slope index of inequality (SII) and relative concentration index (RCI) were used to quantify absolute and relative inequalities across countries ranked by gross domestic product (GDP).

**Results:**

Between 1990 and 2022, global overweight prevalence among children and adolescents approximately doubled, from 5.76% to 11.50%, with an AAPC of 2.18%, whereas obesity prevalence increased more than fourfold, from 1.93% to 8.22%, with an AAPC of 4.62%. By 2040, overweight and obesity prevalence are projected to reach 15.68% and 13.57%, respectively, with corresponding AAPCs of 1.66% and 2.76% during 2023–2040. From 1990 to 2040, overweight and obesity rates rose across all income groups, fastest in low- and middle-income countries and slower in high-income countries (HICs). SII estimates show that overweight disparities between lower- and higher-income countries declined from 15.27% in 1990 to 12.81% in 2022, and then to 9.32% by 2040, while obesity disparities increased from 6.71% to 13.80%, and then to 12.30%. The RCI declined for both outcomes, indicating a reduction in their relative concentration in higher-income countries over time.

**Conclusions:**

The global burden of overweight and obesity rose substantially from 1990 to 2022 and is projected to continue increasing through 2040, with the fastest projected growth in low- and middle-income countries and slower growth in HICs. Although the burdens of overweight and obesity remain disproportionately concentrated in higher-income countries, the degree of relative health inequality has gradually diminished over time.

## 1 Introduction

The increasing prevalence of excess weight among individuals aged 5 to 19 years poses a growing challenge to population health and healthcare systems worldwide. From 1975 to 2016, global age-standardised obesity prevalence among individuals aged 5–19 years rose from 0.7% to 5.6% in girls and from 0.9% to 7.8% in boys (1–3). In developing countries, the combined prevalence of overweight and obesity increased from 8.1% to 12.9% in boys and from 8.4% to 13.4% in girls between 1980 and 2013 (4). While the rates of overweight and obesity have plateaued at high levels in many high-income countries, they continue to accelerate in low- and middle-income countries, particularly in regions of Asia and Africa (1). Importantly, this ongoing upward trend emerges against a backdrop of persistent undernutrition, resulting in the coexistence of nutritional deficiency and excess body weight in many resource-limited regions (5). These trends are concerning because childhood and adolescent overweight and obesity are linked to immediate and long-term health consequences, including metabolic disorders (6–8), cardiovascular risk factors (9), psychological effects (10), and increased healthcare costs (11). Childhood overweight and obesity impose a substantial economic burden, adding an estimated US$237.55 in annual medical costs per affected child compared with healthy weight (11). By 2050, annual global direct and indirect costs are projected to reach US$13.62 billion and US$49.02 billion, respectively; all estimates are expressed in 2022 US dollars (11).

The growing prevalence of overweight and obesity in children and adolescents reflects the complex interplay of genetic predisposition, individual behaviours, environmental exposures, and broader socio-cultural and economic contexts (2, 12). Changes in dietary habits toward high-calorie, low-nutrient foods; fewer opportunities for physical activity due to urbanisation and increased use of digital devices; and more sedentary behaviour, particularly prolonged screen time, have been recognized as key factors driving this epidemic (13). However, these individual behaviours are also shaped by broader social, structural, and commercial determinants. Aggressive marketing of energy-dense, nutrient-poor foods to children, the relatively high prices and limited accessibility of healthy foods, the widespread availability and affordability of ultra-processed foods, and unequal school and neighbourhood food environments may promote unhealthy dietary patterns and restrict opportunities for healthier choices (14–17). These factors often disproportionately affect children in disadvantaged communities, thereby reinforcing and amplifying existing health disparities (18).

Recognizing these challenges, the WHO Commission on Ending Childhood Obesity has emphasized the need for multisectoral and equity-oriented interventions beginning early in life (19, 20). NCD-RisC has documented global trends in underweight and obesity among children and adolescents through 2022 (3), while a previous analysis projected adult overweight and obesity prevalence to 2040 and assessed cross-country socioeconomic inequalities (21). However, adult patterns cannot be directly extrapolated to younger populations because of age-specific BMI definitions, developmental trajectories, distinct exposure contexts, and different opportunities for prevention. The present study therefore examined global, regional, and national trends in overweight and obesity among children and adolescents aged 5–19 years from 1990 to 2022, projected their prevalence through 2040, and quantified the magnitude and temporal changes in health inequalities in the distribution of these burdens across countries at different levels of economic development.

## 2 Methods

### 2.1 Data source

Country-, year-, age-, and sex-specific prevalence estimates of overweight and obesity were obtained from NCD-RisC, which synthesised measured height and weight data from 3,663 population-representative studies involving approximately 222 million participants. We extracted estimates for individuals aged 5–19 years across 200 countries and territories from 1990 to 2022. Details of the underlying data sources and the Bayesian hierarchical model used by NCD-RisC have been reported previously (1, 3). Overweight and obesity were defined according to the WHO growth reference for school-aged children and adolescents, with overweight corresponding to a BMI >1 standard deviation (SD) and ≤2 SD above the age- and sex-specific median, and obesity defined as a BMI >2 SD above the median (1). Countries and territories were classified as low-income countries (LICs), lower-middle-income countries (LMICs), upper-middle-income countries (UMICs), and high-income countries (HICs) according to the World Bank income classification (22).

### 2.2 Temporal analysis of overweight and obesity prevalence

To examine temporal trends in prevalence, a log-linear regression framework was applied. Temporal patterns were further analysed using Joinpoint regression, a segmented regression technique designed to detect statistically significant changes in trend. A grid search algorithm was implemented to identify optimal joinpoints and segment the study period into distinct intervals.

For each interval, trend estimation and optimisation were conducted independently, facilitating a detailed assessment of temporal dynamics across varying time segments. The robustness of the model selection process was ensured through Monte Carlo permutation testing. The average annual percentage change (AAPC) was used to summarise the overall temporal trend across the entire study period.

### 2.3 Bayesian age-period-cohort model for forecasting overweight and obesity prevalence

This study used Bayesian age-period-cohort (BAPC) models (23) to project the prevalence of overweight and obesity among children and adolescents aged 5–19 years from 2023 to 2040, based on annual data from 1990 to 2022. The models jointly accounted for age, period, and birth cohort effects to characterise historical patterns and project future changes. Analyses were conducted separately by country or territory, sex, outcome, and three 5-year age groups (5–9, 10–14, and 15–19 years).

Bayesian posterior inference was performed using integrated nested Laplace approximation through the BAPC and INLA packages in R. Projections were summarised using posterior means and approximate 95% CI. Temporal holdout validation was conducted by fitting the models using data from 1990 to 2012 and predicting prevalence from 2013 to 2022. Predictive accuracy was evaluated by comparing the predicted estimates with the corresponding NCD-RisC reference estimates using the mean absolute error and root mean squared error, both expressed in percentage points. Details of the model specification, prior distributions, forecasting procedure, and validation metrics are provided in the **Supplementary Methods** section of the Appendix.

### 2.4 Cross-country health inequalities analysis

Cross-country inequalities in childhood and adolescent overweight and obesity were assessed following the standard health inequality monitoring framework implemented in the World Health Organization’s Health Equity Assessment Toolkit (HEAT/HEAT Plus). We used the slope index of inequality (SII) and the relative concentration index (RCI) to quantify absolute and relative Gross Domestic Product (GDP)-related inequalities, respectively, in the prevalence of overweight and obesity among children and adolescents aged 5–19 years.

For each analysis year, countries and territories were ranked from lowest to highest GDP per capita and assigned a ridit score corresponding to the midpoint of their cumulative share of the population aged 5 to 19 years. For overweight and obesity separately, prevalence estimates were regressed on the logarithmically transformed ridit score using a weighted regression model to account for heteroskedasticity and potential nonlinearity. The SII was defined as the model-predicted prevalence at the highest position of the GDP per capita distribution minus that at the lowest position. The RCI was calculated using the corresponding concentration curve and covariance-based estimator to assess the relative concentration of the burden across the full socioeconomic distribution. Positive SII and RCI values indicate a greater burden in countries with higher GDP per capita, whereas negative values indicate a greater burden in countries with lower GDP per capita.

### 2.5 Statistical analysis

Data processing, BAPC modelling, and cross-country health inequality analyses were conducted using R version 4.2.3. BAPC models were implemented using the BAPC package (version 0.0.36) and the INLA package (version 23.9.9) in R, while AAPCs were estimated using the Joinpoint Regression Program (version 4.9.0.0).

## 3 Results

### 3.1 Model validation

During the 2013–2022 validation period, the BAPC-predicted prevalence estimates for overweight and obesity were generally close to the corresponding NCD-RisC reference estimates. Comparisons between the predicted and reference estimates for each validation year are presented in **Supplementary Figure 1**. The mean MAE and RMSE were 0.31% and 0.42%, respectively, for overweight prevalence, and 0.47% and 0.61%, respectively, for obesity prevalence.

### 3.2 Global trends of overweight and obesity among children and adolescents

Globally, the overweight prevalence among children and adolescents increased from 5.76% (95% CI, 4.01%–7.51%) in 1990 to 11.50% (7.88%–15.12%) in 2022 and is projected to reach 15.68% (12.83%–18.54%) by 2040. The corresponding average annual percentage change (AAPC) was 2.18% (95% CI, 2.15%–2.20%) during 1990–2022 and 1.66% (1.64%–1.69%) during the projected period of 2023–2040 (**Table 1**).

**Table 1:** Prevalence and number of cases of overweight and obesity, and average annual percentage changes (AAPCs) in their prevalence, among children and adolescents aged 5 to 19 years at global, income-group, and regional levels, from 1990 to 2040.

Characteristics	1990		2022		2040		1990-2022	2023-2040
	Cases million (95% CI)	Prevalence, % (95% CI)	Cases million (95% CI)	Prevalence, % (95% CI)	Cases million (95% CI)	Prevalence, % (95% CI)	AAPC, % (95% CI)	AAPC, % (95% CI)
Overweight								
Global	94(65–122)	5.76(4.01–7.51)	230(158–303)	11.50(7.88–15.12)	315(258–373)	15.68(12.83–18.54)	2.18(2.15–2.20)	1.66(1.64–1.69)
Country income groups								
HICs	32(25–38)	14.37(11.48–17.27)	38(30–47)	18.55(14.29–22.81)	35(29–42)	20.11(16.67–23.56)	0.80(0.78–0.82)	0.41(0.4–0.42)
UMICs	39(28–50)	6.08(4.34–7.81)	85(63–106)	14.42(10.78–18.05)	91(74–107)	21.65(17.70–25.60)	2.73(2.68–2.78)	2.12(2.04–2.20)
LMICs	19(11–28)	2.94(1.62–4.26)	85(55–115)	9.05(5.88–12.21)	137(110–163)	13.66(10.99–16.32)	3.59(3.56–3.62)	2.28(2.26–2.31)
LICs	4(1–6)	3.31(1.24–5.37)	22(10–35)	8.27(3.58–12.95)	53(45–61)	12.81(10.87–14.75)	2.91(2.89–2.94)	2.45(2.43–2.47)
Region								
Africa	6(3–10)	3.31(1.32–5.29)	35(17–53)	7.84(3.78–11.91)	71(59–83)	11.11(9.18–13.04)	2.74(2.72–2.76)	1.94(1.93–1.95)
Americas	27(20–34)	12.98(9.68–16.31)	47(37–57)	20.63(16.23–25.03)	47(40–54)	23.26(19.77–26.75)	1.45(1.44–1.47)	0.67(0.66–0.67)
Europe	25(18–32)	12.99(9.16–16.82)	27(19–34)	15.83(11.44–20.23)	24(19–29)	16.67(13.04–20.30)	0.62(0.61–0.63)	0.29(0.28–0.30)
Eastern Mediterranean	11(7–15)	7.74(4.64–10.85)	37(23–51)	14.70(9.16–20.24)	63(52–74)	20.31(16.76–23.86)	2.03(2.01–2.06)	1.79(1.78–1.80)
South-East Asia	6(3–8)	1.23(0.71–1.76)	43(31–55)	7.88(5.64–10.11)	68(53–83)	13.65(10.68–16.62)	5.98(5.94–6.03)	3.06(3.03–3.09)
Western Pacific	19(15–22)	4.26(3.45–5.06)	42(31–53)	11.42(8.43–14.41)	42(35–49)	19.5(16.28–22.71)	3.13(3.08–3.17)	2.63(2.44–2.83)
Obesity								
**Global**	31(18–45)	1.93(1.10–2.77)	165(99–230)	8.22(4.95–11.49)	273(208–338)	13.57(10.34–16.81)	4.62(4.59–4.65)	2.76(2.74–2.79)
Country income groups								
HICs	13(9–17)	5.87(4.13–7.61)	28(19–37)	13.56(9.4–17.72)	29(19–39)	17.05(11.70–22.40)	2.64(2.6–2.69)	1.24(1.22–1.26)
UMICs	12(7–17)	1.82(1.01–2.64)	72(50–95)	12.36(8.54–16.19)	92(71–113)	21.95(17.00–26.90)	6.16(6.09–6.23)	3.20(3.17–3.23)
LMICs	6(2–10)	0.90(0.32–1.47)	52(27–76)	5.49(2.85–8.13)	111(86–137)	11.11(8.57–13.66)	5.82(5.79–5.84)	3.92(3.87–3.96)
LICs	1(0–2)	0.82(0.12–1.53)	12(3–22)	4.60(1.04–8.15)	41(32–50)	9.93(7.70–12.17)	5.51(5.48–5.54)	4.31(4.24–4.38)
Region								
Africa	1(0–3)	0.74(0.12–1.36)	18(5–30)	3.98(1.12–6.83)	51(38–64)	7.99(5.96–10.03)	5.42(5.33–5.51)	3.88(3.83–3.93)
Americas	12(8–16)	5.57(3.65–7.48)	39(28–49)	16.98(12.25–21.71)	47(36–58)	23.41(18.04–28.77)	3.54(3.50–3.57)	1.78(1.77–1.79)
Europe	7(4–11)	3.80(2.03–5.57)	13(8–19)	7.94(4.66–11.22)	14(9–19)	9.73(6.08–13.37)	2.33(2.31–2.36)	1.14(1.10–1.17)
Eastern Mediterranean	4(2–7)	3.12(1.29–4.94)	30(15–45)	11.89(6.09–17.7)	69(54–83)	22.21(17.61–26.81)	4.28(4.24–4.31)	3.47(3.43–3.52)
South-East Asia	2(1–2)	0.35(0.14–0.55)	25(15–35)	4.62(2.78–6.46)	49(37–61)	9.82(7.47–12.18)	8.45(8.37–8.53)	4.20(4.13–4.26)
Western Pacific	5(4–7)	1.17(0.83–1.52)	40(28–52)	10.8(7.57–14.03)	43(33–53)	19.72(15.14–24.3)	7.16(7.07–7.26)	3.33(3.28–3.38)

AAPC, Average annual percentage change; LICs, low-income countries; LMICs, lower-middle-income countries; UMICs, upper-middle-income countries; HICs, high-income countries.

Similarly, the global obesity prevalence increased more rapidly, rising from 1.93% (1.10%–2.77%) in 1990 to 8.22% (4.95%–11.49%) in 2022, with a further increase to 13.57% (10.34%–16.81%) projected by 2040. The AAPC was 4.62% (4.59%–4.65%) during 1990–2022 and 2.76% (2.74%–2.79%) during 2023–2040 (**Table 1**).

### 3.3 Global trends of overweight and obesity among children and adolescents by income groups

From 1990 to 2040, overweight prevalence has steadily increased, especially in UMICs, LMICs, and LICs. Among these, the most rapid growth occurred in LMICs, where prevalence rose from 2.94% (1.62%–4.26%) in 1990 to 9.05% (5.88%–12.21%) in 2022, corresponding to an AAPC of 3.59% (3.56%–3.62%). This increase is projected to continue, although at a slower annual rate, with prevalence reaching 13.66% (10.99%–16.32%) by 2040 and an AAPC of 2.28% (2.26%–2.31%) during 2023–2040. In contrast, HICs experienced the slowest growth, with prevalence increasing from 14.37% (11.48%–17.27%) in 1990 to 18.55% (14.29%–22.81%) in 2022 (AAPC = 0.80% [0.78%–0.82%]), and a modest rise projected to 20.11% (16.67%–23.56%) by 2040, with an AAPC of 0.41% (0.40%–0.42%) (**Table 1**).

Obesity prevalence increased across all income groups from 1990 to 2022, with further increases projected through 2040, with more rapid growth seen in low- and middle-income countries (UMICs, LMICs, and LICs). Among these, UMICs showed the largest rise, with obesity prevalence increasing 6.8-fold, from 1.82% (1.01%–2.64%) in 1990 to 12.36% (8.54%–16.19%) in 2022 (AAPC = 6.16% [6.09%–6.23%]), and projected to reach 21.95% (17.00%–26.90%) by 2040 (AAPC = 3.20% [3.17%–3.23%]). LMICs and LICs also followed a similar pattern of rapid increase. In contrast, HICs experienced slower growth, with prevalence rising from 5.87% (4.13%–7.61%) in 1990 to 13.56% (9.40%–17.72%) in 2022 (AAPC = 2.64% [2.60%–2.69%]), and expected to reach 17.05% (11.70%–22.40%) by 2040, with a slower pace of change (AAPC = 1.24% [1.22%–1.26%]) (**Table 1**).

### 3.4 Global trends of overweight and obesity by geographical regions among children and adolescents

Regional trajectories differed considerably during the observed and projected periods, despite increases in both outcomes across all six WHO regions (**Table 1**). In 1990, the population-weighted prevalence of overweight was highest in Europe and the Americas, at approximately 13% in both regions. In contrast, the corresponding prevalence in the Western Pacific was substantially lower, at 4.26%. However, marked within-region heterogeneity was evident, with estimated prevalence already exceeding 10%, and in some cases 20%, in several Pacific island countries and territories. Because the regional estimate was population weighted, the relatively low prevalence in highly populous countries, particularly China, had a greater influence on the regional average (**Table 1**; **Supplementary Figure 2A**). An estimated overweight prevalence above 20% was recorded in 44 countries and territories in 2022, representing 22.0% of all locations analysed; most were situated in the Americas, Eastern Mediterranean, or Western Pacific (**Supplementary Table 1**; **Figures 1A**, **2A**). At the opposite end of the distribution, prevalence remained below 10% in 48 locations (24.0%), which were concentrated largely in Sub-Saharan Africa. The geographical extent of high overweight prevalence is projected to expand substantially by 2040, when 81 locations (40.5%) are expected to exceed the 20% threshold. These locations are projected to be concentrated chiefly in the Americas, Eastern Mediterranean, and Western Pacific (**Supplementary Table 1**; **Figure 2B**). Only 18 locations (9.0%) are expected to remain below 10% in 2040, with most of these located in Sub-Saharan Africa. The maps also show the country-level distributions of the observed AAPCs in overweight prevalence during 1990–2022 and the projected AAPCs during 2023–2040 (**Figures 2C, D**).

**Figure 1:**
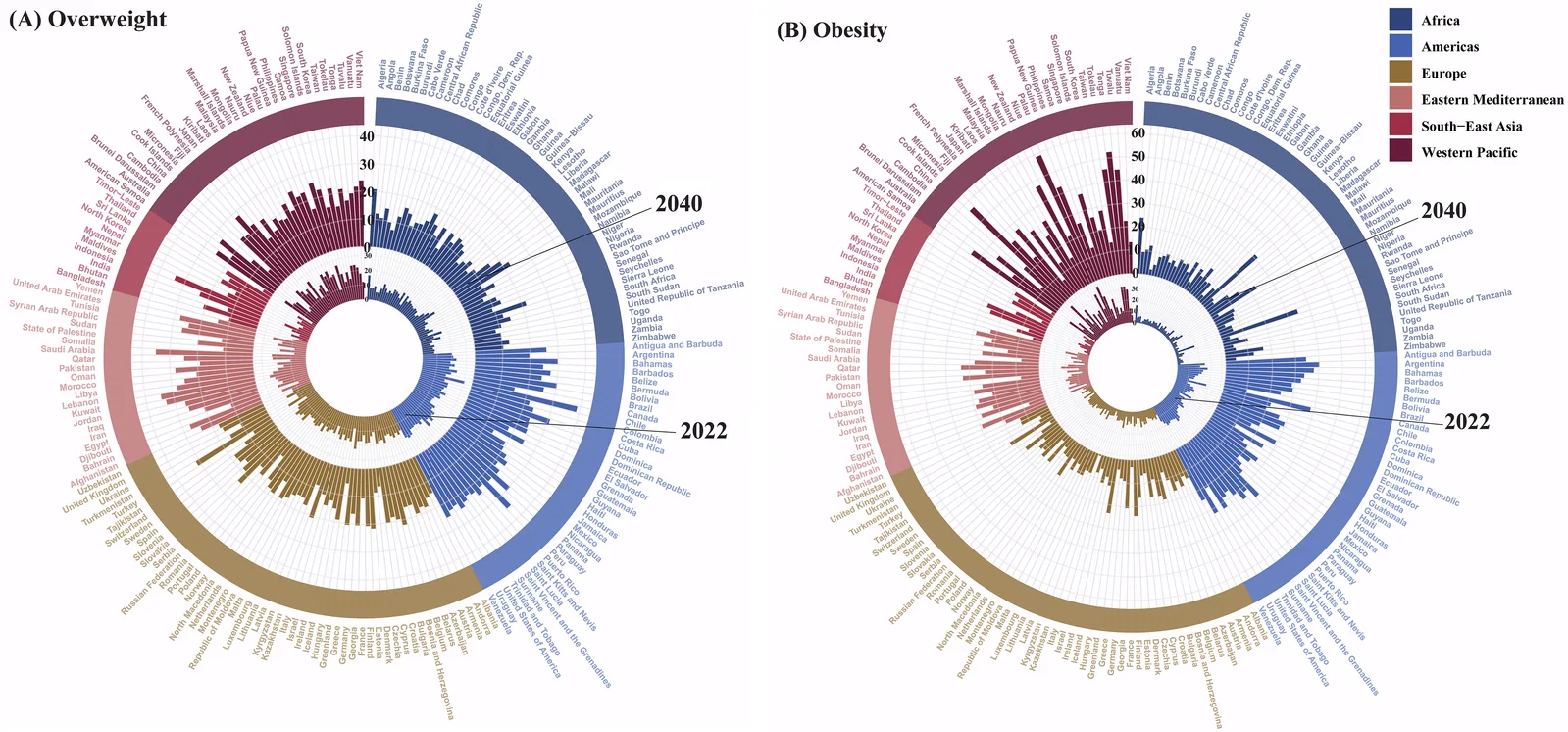
Prevalence of overweight **(A)** and obesity **(B)** by country and region in 2022 and 2040 among children and adolescents (aged 5–19 years).

**Figure 2:**
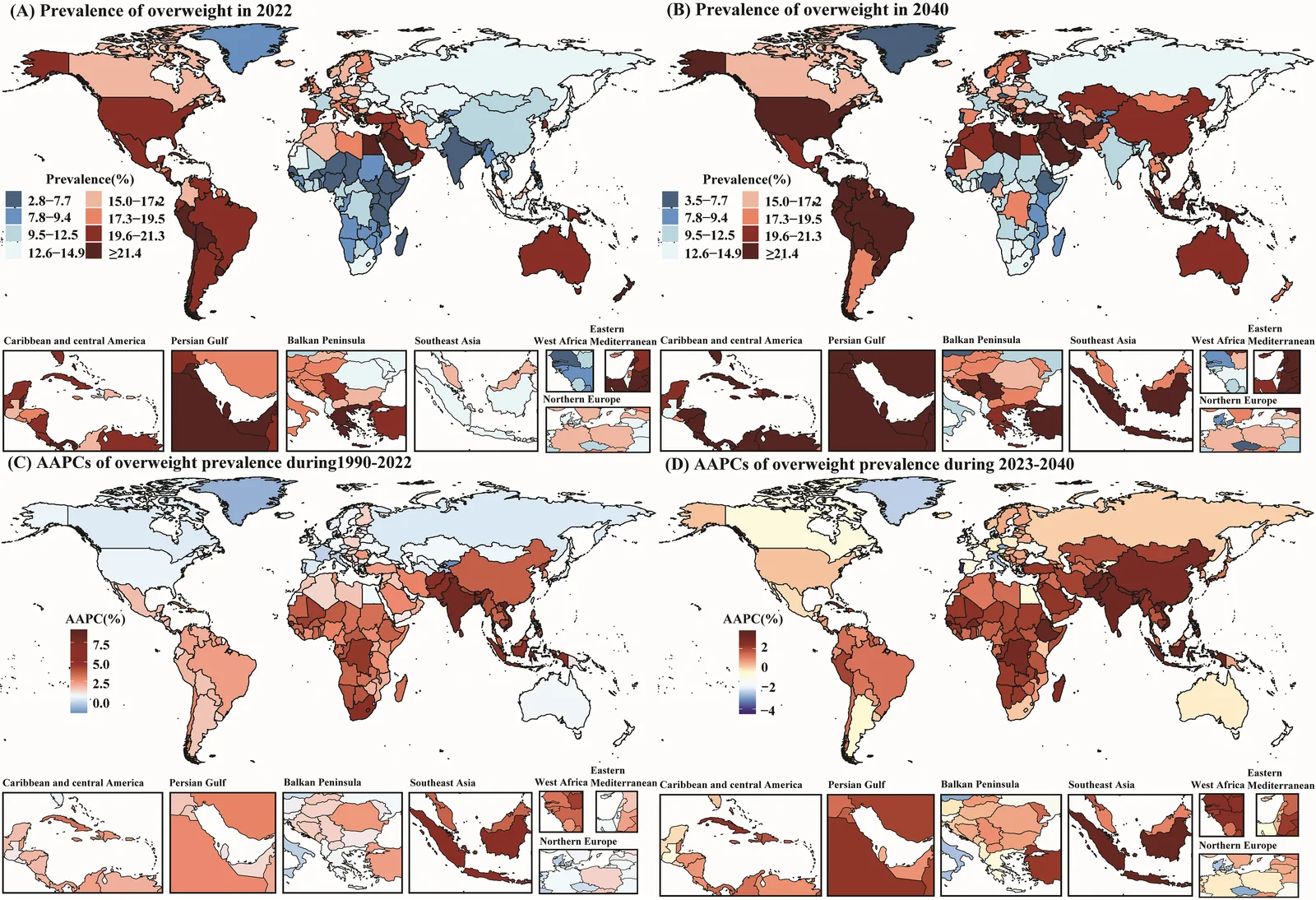
Global distribution of overweight prevalence in 2022 **(A)** and 2040 **(B)**, with corresponding average annual percentage change (AAPC) from 1990 to 2022 **(C)** and from 2023 to 2040 **(D)** among children and adolescents (aged 5–19 years) by country and territory.

In 1990, obesity prevalence was highest in the Americas at 5.57%, followed by Europe at 3.80%, whereas prevalence remained below 1% in Africa and South-East Asia (**Table 1**; **Supplementary Figure 2B**). In 2022, the 20% obesity threshold was exceeded in 26 locations, accounting for 13.0% of the 200 countries and territories analysed. These locations were concentrated in the Americas, Eastern Mediterranean, and Western Pacific (**Supplementary Table 2**; **Figures 1B**, **3A**). Prevalence remained below 5% in 45 locations (22.5%), the majority of which were situated in Sub-Saharan Africa (**Supplementary Table 2**; **Figures 1B**, **3A**). By 2040, obesity prevalence is projected to exceed 20% in 81 countries and territories, representing 40.5% of all locations analysed and more than three times the number in 2022, with most located in the Americas, Eastern Mediterranean, and Western Pacific regions (**Supplementary Table 2**; **Figure 3B**). Conversely, only 22 locations (11.0%) are projected to remain below 5%, primarily in Africa and parts of Europe (**Supplementary Table 2**; **Figure 3B**). The maps also show the country-level distributions of the observed AAPCs in obesity prevalence during 1990–2022 and the projected AAPCs during 2023–2040 (**Figures 3C, D**).

**Figure 3:**
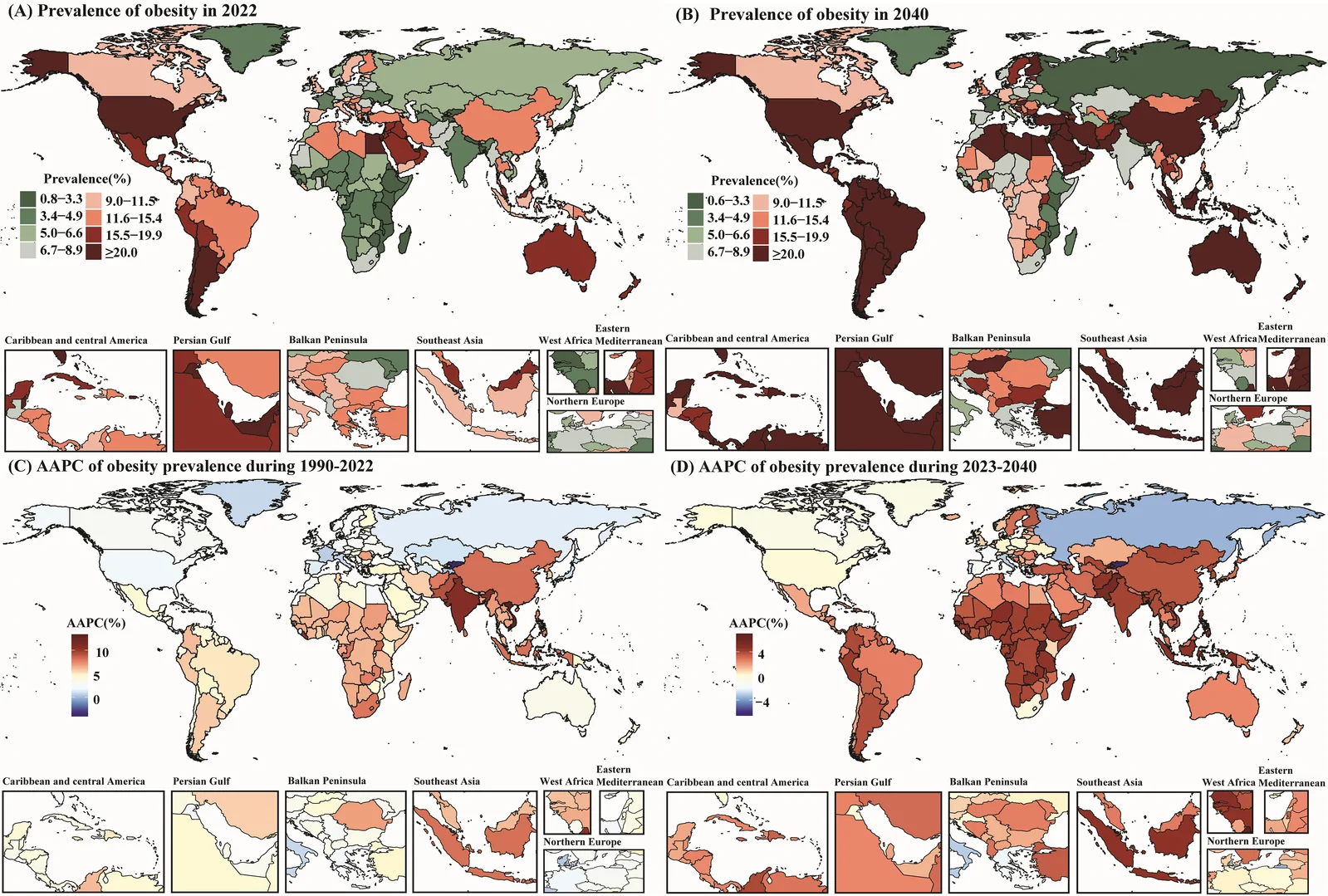
Global distribution of obesity prevalence in 2022 **(A)** and 2040 **(B)**, with corresponding average annual percentage change (AAPC) from 1990 to 2022 **(C)** and from 2023 to 2040 **(D)** among children and adolescents (aged 5–19 years) by country and territory.

### 3.5 Health inequalities of overweight and obesity among children and adolescents

We identified substantial socioeconomic inequalities in childhood and adolescent overweight and obesity prevalence, as measured by both absolute and relative gradient indices, with both burdens disproportionately concentrated in countries with higher GDP per capita. The SII for global overweight prevalence revealed an absolute difference of 15.27% (15.15%–15.39%) between countries with the lowest and highest GDP per capita in 1990 (**Figure 4A**). The positive value indicated that overweight prevalence was disproportionately concentrated in countries with higher GDP per capita. By 2022, this absolute inequality had declined to 12.81% (12.76%–12.86%), and it is projected to decrease further to 9.32% (9.31%–9.33%) by 2040 (**Figure 4A**). Similarly, the RCI decreased from 39.71% (37.80%–41.63%) in 1990 to 18.11% (17.01%–19.21%) in 2022 and is projected to decline further to 10.58% (10.01%–11.15%) by 2040 (**Figure 4B**). Consistently, both indices indicate a progressive reduction in the absolute and relative socioeconomic inequalities in overweight prevalence, although the burden remains relatively concentrated in countries with higher GDP per capita.

**Figure 4:**
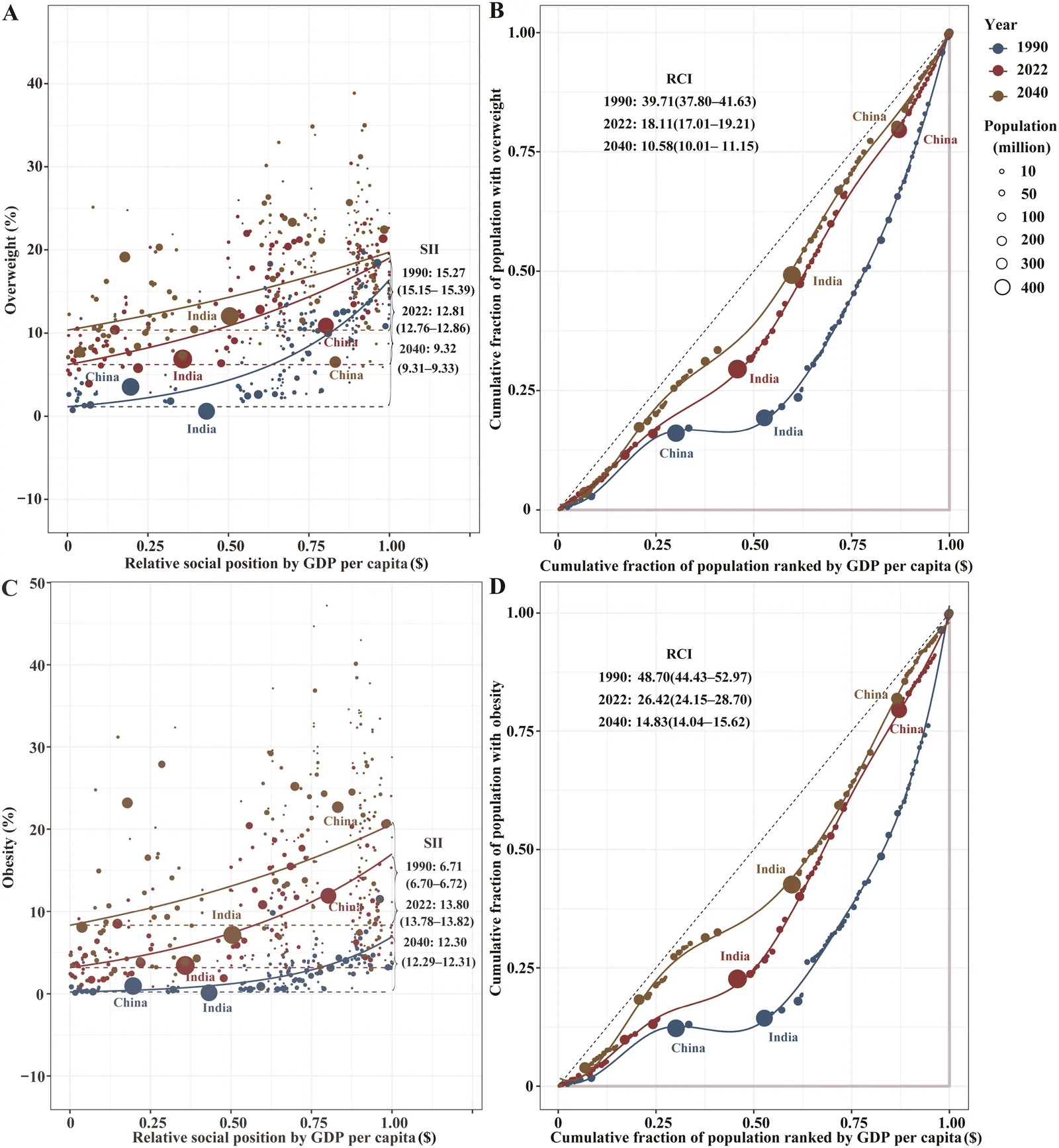
GDP per capita-related health inequality analysis for overweight **(A, B)** and obesity **(C, D)** prevalence among children and adolescents (aged 5–19 years) by country or territory (1990, 2022, 2040) **(A, C)** Absolute inequality: GDP per capita-related regression curves with slope index of inequality (SII, 95% CI); **(B, D)** Relative inequality: GDP per capita-related concentration curves with relative concentration index (RCI, 95% CI).

For obesity prevalence, the SII demonstrated an absolute difference of 6.71% (6.70%–6.72%) between countries with the lowest and highest GDP per capita in 1990 (**Figure 4C**). This absolute inequality widened to 13.80% (13.78%–13.82%) by 2022 and is projected to decrease slightly to 12.30% (12.29%–12.31%) by 2040, while remaining substantially greater than the level observed in 1990 (**Figure 4C**). In contrast, the RCI declined markedly from 48.70% (44.43%–52.97%) in 1990 to a projected 14.83% (14.04%–15.62%) in 2040 (**Figure 4D**). The positive RCI values indicate that obesity prevalence remained relatively concentrated in countries with higher GDP per capita, whereas the declining magnitude indicates that this relative concentration weakened over time.

## 4 Discussion

Both overweight and obesity prevalence increased substantially during 1990–2022 and are projected to rise further through 2040. Overweight prevalence is expected to increase from 11.50% in 2022 to 15.68% in 2040, whereas obesity prevalence is projected to rise more steeply from 8.22% to 13.57%. Although increases were observed across all income groups, they were more pronounced in LICs, LMICs, and UMICs than in HICs. Upward trends were also widespread geographically, with the steepest increases observed in Sub-Saharan Africa, South Asia, and Southeast Asia. Despite the continued disproportionate concentration of overweight and obesity burdens in countries with higher GDP per capita between 1990 and 2040, the magnitude of relative inequality declined progressively over time.

The faster increase in obesity than in overweight among children and adolescents may reflect the combined influence of behavioural, psychosocial, socioeconomic, and early-life factors (2, 24). Previous studies have associated greater consumption of sugar-sweetened beverages, ultra-processed foods, and fast foods, together with lower levels of physical activity and more sedentary behaviour, with a higher risk of excess weight gain (25–28). However, these behaviours are embedded within broader social conditions. Socioeconomic disadvantage may limit access to healthy foods and safe recreational spaces, while psychosocial stress, inadequate sleep, and mental health problems may affect appetite regulation and metabolic health (28). Prenatal nutrition and other early-life exposures may also influence metabolic development and later susceptibility to obesity (29). In addition, childhood obesity is associated with stigma, emotional distress, and impaired quality of life (30).

The rapid increases projected for Sub-Saharan Africa, South Asia, and Southeast Asia are particularly concerning because overweight and obesity are rising in settings where undernutrition often remains prevalent, creating a double burden of malnutrition (5). Early-life undernutrition may alter growth trajectories and metabolic development, potentially increasing later susceptibility to adiposity, insulin resistance, and other cardiometabolic disorders (31, 32). These overlapping risks support life-course strategies that address maternal and child undernutrition while also preventing excess weight gain (33). The rapid changes observed in many low- and middle-income countries are also consistent with a compressed nutrition transition occurring over a relatively short period. The expansion of global food supply chains and the increased availability of ultra-processed foods may contribute to changes in local food environments (34, 35). Marketing directed at children and adolescents may further shape food preferences and consumption (17, 36). Nevertheless, these contextual factors should not be interpreted as causal mechanisms established by the present country-level analysis. In comparison, childhood obesity prevalence has plateaued or increased more slowly in some high-income countries, but these trends cannot be attributed to any single policy, intervention, or socioeconomic change.

A particularly important finding is the projected crossover in childhood and adolescent obesity prevalence between UMICs and HICs. By 2040, obesity prevalence is projected to reach 21.95% in UMICs, exceeding the corresponding estimate of 17.05% in HICs. This finding indicates an important shift in the global distribution of childhood obesity and suggests that UMICs may face an especially substantial public health challenge. Rapid increases in prevalence may outpace the development of prevention, early detection, and treatment services in these settings. Responses should therefore move beyond stand-alone health education and prioritise coordinated, and multilevel strategies. These may include enforceable nutrition standards for foods and beverages provided or sold in and around schools, protection and promotion of breastfeeding, restrictions on the marketing of energy-dense and nutrient-poor products to children, improved access to healthy foods and opportunities for physical activity, and accessible services for the early identification and management of childhood obesity (14, 15, 37–39). Evidence from some HIC settings suggests that sustained, multicomponent whole-systems approaches spanning policy, school, community, and healthcare settings may contribute to improvements in weight-related outcomes (40, 41).

Our findings indicate that overweight and obesity remained disproportionately concentrated in countries with higher GDP per capita during the observed period and are projected to remain so through 2040. Although the relative concentration of overweight and obesity in higher-income countries declined over time, absolute inequality in obesity remained substantially greater than in 1990. This pattern indicates a convergence in the relative distribution of childhood and adolescent overweight and obesity across countries at different income levels, rather than the elimination of socioeconomic inequality. This shift in the global distribution of childhood and adolescent obesity underscores the need for international health cooperation, particularly to support low-resource settings in implementing effective preventive strategies (2). Without targeted and culturally appropriate interventions, low- and middle-income countries may face obesity burdens comparable to those in HICs without having similarly developed capacity for prevention, early detection, and treatment. This possibility highlights the need for coordinated early life, school based, and policy level strategies tailored to local socioeconomic conditions, health system capacity, and cultural contexts. Taken together, the integration of historical trends, future projections, and socioeconomic inequality measures may help inform evidence based public health policies that prioritise equity in child health and support targeted and inclusive strategies to prevent childhood overweight and obesity and reduce their long term noncommunicable disease consequences.

This study has several strengths. Using the NCD-RisC database, we integrated historical trends in the prevalence of childhood and adolescent overweight and obesity from 1990 to 2022, projections to 2040, and absolute and relative socioeconomic inequalities quantified using the SII and RCI within a single analytical framework. This integrated framework allowed us not only to characterise past changes and projected future trajectories in prevalence, but also to assess how the socioeconomic distribution of these burdens changed over time. Our study also has several limitations. First, NCD-RisC estimates for locations and age groups with sparse nationally representative data rely more heavily on information borrowing across locations and time; uncertainty may therefore be greater for these settings, particularly where data for children aged 5–9 years are limited. Second, BMI-for-age is an indirect measure of adiposity and does not distinguish fat mass from lean mass or fully capture population-specific variation in body composition and metabolic risk. Third, this ecological analysis characterises inequality across national economic positions and should not be interpreted as evidence of individual-level socioeconomic associations or causal effects. Finally, the BAPC projections extrapolate historical age, period, and cohort patterns and do not incorporate unforeseen policy, economic, technological, or environmental changes.

In conclusion, the global burden of overweight and obesity increased substantially from 1990 to 2022 and is projected to continue increasing through 2040. Increases were most substantial in low- and middle-income countries, whereas the corresponding changes in high-income countries were comparatively smaller. Growth is expected to be most rapid in regions including Sub-Saharan Africa, South Asia, and Southeast Asia. Although the burdens of overweight and obesity remain disproportionately concentrated in higher-income countries, the degree of relative inequality has gradually diminished over time, reflecting the increasingly widespread distribution of these burdens across low- and middle-income countries. These findings highlight the need for sustained and equity-oriented strategies across all economic settings. Specific policy priorities should include enforceable nutrition standards for foods and beverages provided or sold in schools and fiscal measures such as taxation of sugar-sweetened beverages, with implementation adapted to local socioeconomic and health-system contexts.

## Data Availability

The original contributions presented in the study are included in the article and Supplementary Material. The data used in this study are publicly available from the NCD Risk Factor Collaboration (NCD-RisC) at https://www.ncdrisc.org/index.html. We gratefully acknowledge NCD-RisC and its contributors for making these data publicly available. Further inquiries can be directed to the corresponding author.
