# Supplementary material for: Global trends, projections, and inequalities in overweight and obesity among children and adolescents from 1990 to 2040: a modelling study
Source: Front Endocrinol (Lausanne). 2026 Sep 22;17:1855271. doi: 10.3389/fendo.2026.1855271 (PMC13638365; doi:10.3389/fendo.2026.1855271)
Supplement: Supplementary file 1 [file DataSheet1.pdf]

Supplementary Materials：

Global trends, projections, and inequalities in overweight and obesity among children and adolescents from 1990 to 2040: a modelling study

This appendix provides additional methodological details and supplementary results.

Contents

1. Supplementary Methods……2

1.1 Bayesian age-period-cohort model for forecasting overweight and obesity prevalence……2

2. Supplementary Figures……3

Supplementary Figure S1. Comparison of BAPC-predicted and NCD-RisC reference prevalence estimates during the 2013–2022 validation period……3

Supplementary Figure S2. Prevalence of overweight (A) and obesity (B) by country and region in 1990 among children and adolescents (age 5–19 years) ……4

3. Supplementary Tables……5

Supplementary Table 1. Prevalence and number of cases of overweight, and average annual percentage changes (AAPCs) in overweight prevalence, among children and adolescents aged 5 to 19 years, by WHO region and country or territory, from 1990 to 2040.……5

Supplementary Table 2. Prevalence and number of cases of obesity, and average annual percentage changes (AAPCs) in obesity prevalence, among children and adolescents aged 5 to 19 years, by WHO region and country or territory, from 1990 to 2040……14

1. Supplementary Methods

1.1 Bayesian age-period-cohort model for forecasting overweight and obesity prevalence

This study employed Bayesian age-period-cohort (BAPC) [1] models to project the prevalence of overweight and obesity among children and adolescents from 2023 to 2040 using annual data from 1990 to 2022. Children and adolescents aged 5–19 years were divided into three 5-year age groups: 5–9, 10–14, and 15–19 years. The models were fitted separately by country, sex, and outcome (overweight or obesity) and simultaneously accounted for age, period, and birth cohort effects to characterize historical patterns and project future trends.

For each age group and calendar year, the number of children and adolescents with overweight or obesity was estimated by multiplying the age-specific prevalence by the corresponding population size. The estimated outcome counts and population data were then arranged into age-by-period matrices. The numbers of individuals with overweight or obesity were modelled using a Poisson distribution, with the corresponding population size specified as the population at risk. As age was grouped into 5-year intervals and calendar period was defined in 1-year intervals, the grid factor was set to 5 when constructing the age–period–cohort data structure. Age, period, and birth cohort effects were modelled using second-order random walks, with log-Gamma priors with parameters of 1 and 0.00005 assigned to their precision parameters. An independent and identically distributed random effect was also included to account for overdispersion, with a log-Gamma prior with parameters of 1 and 0.005 assigned to its precision parameter.

Bayesian posterior inference was performed using integrated nested Laplace approximation through the BAPC and INLA packages in R. Outcome counts for 2023–2040 were set as missing, and annual projections were generated using the corresponding projected population data. Projected estimates were summarized using posterior means and approximate 95% credible intervals calculated from the posterior standard deviations.

To assess predictive performance, temporal holdout validation was conducted. The models were fitted using data from 1990 to 2012 and used to predict prevalence from 2013 to 2022. The predicted estimates were compared with the corresponding reference estimates, and predictive accuracy was evaluated using the mean absolute error (MAE) and root mean square error (RMSE), expressed in percentage points.

Reference:

1. Chen W-Q, Zheng R-S, Zeng H-M. Bayesian age-period-cohort prediction of lung cancer incidence in China. Thorac Cancer 2011; 2(4): 149-55.

2. Supplementary Figures

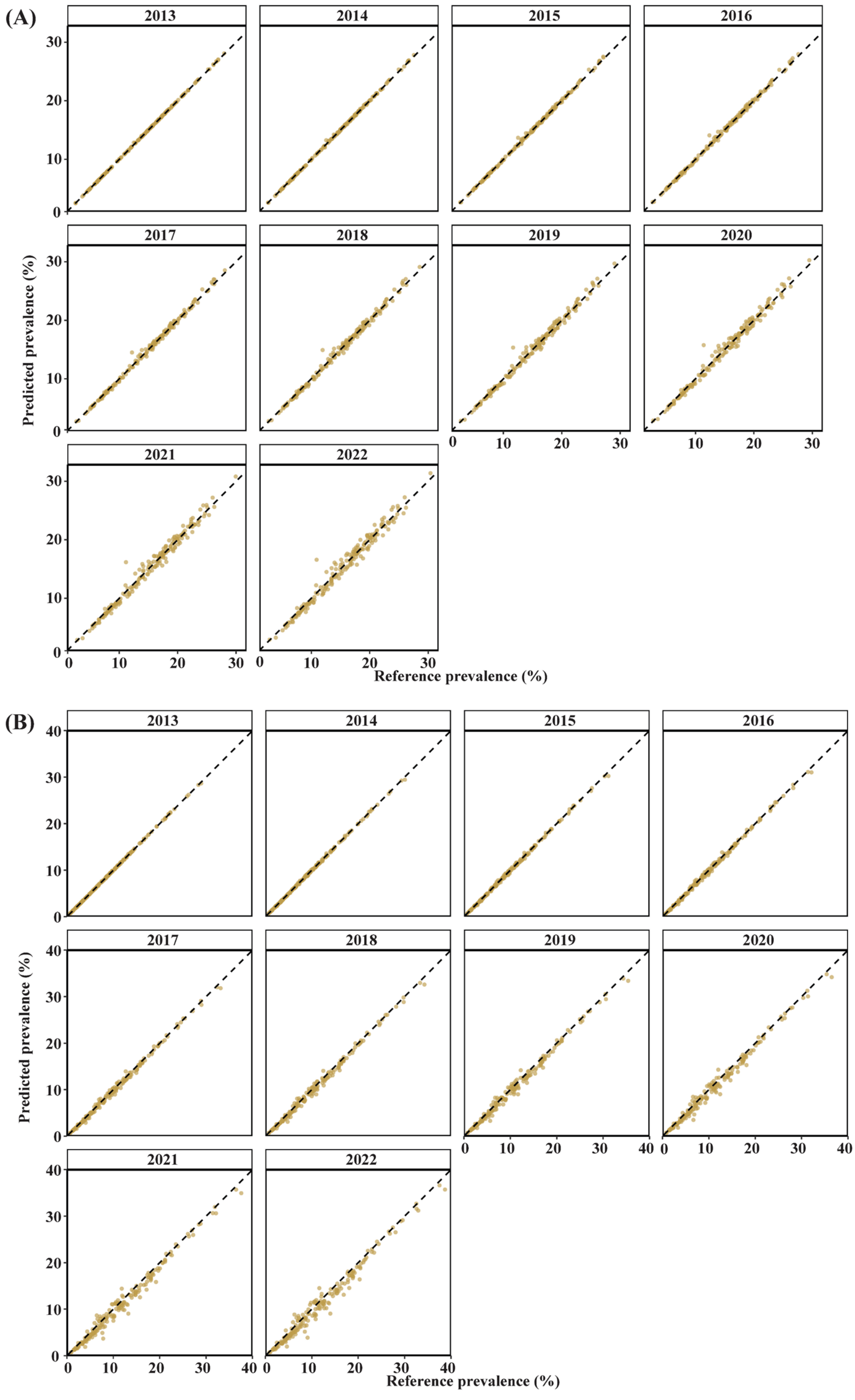

Supplementary Figure S1. Comparison of BAPC-predicted and NCD-RisC reference prevalence estimates during the 2013–2022 validation period. (A) Overweight prevalence; (B) obesity prevalence. Each point represents one country or territory. The dashed diagonal line indicates perfect agreement between the predicted and reference estimates

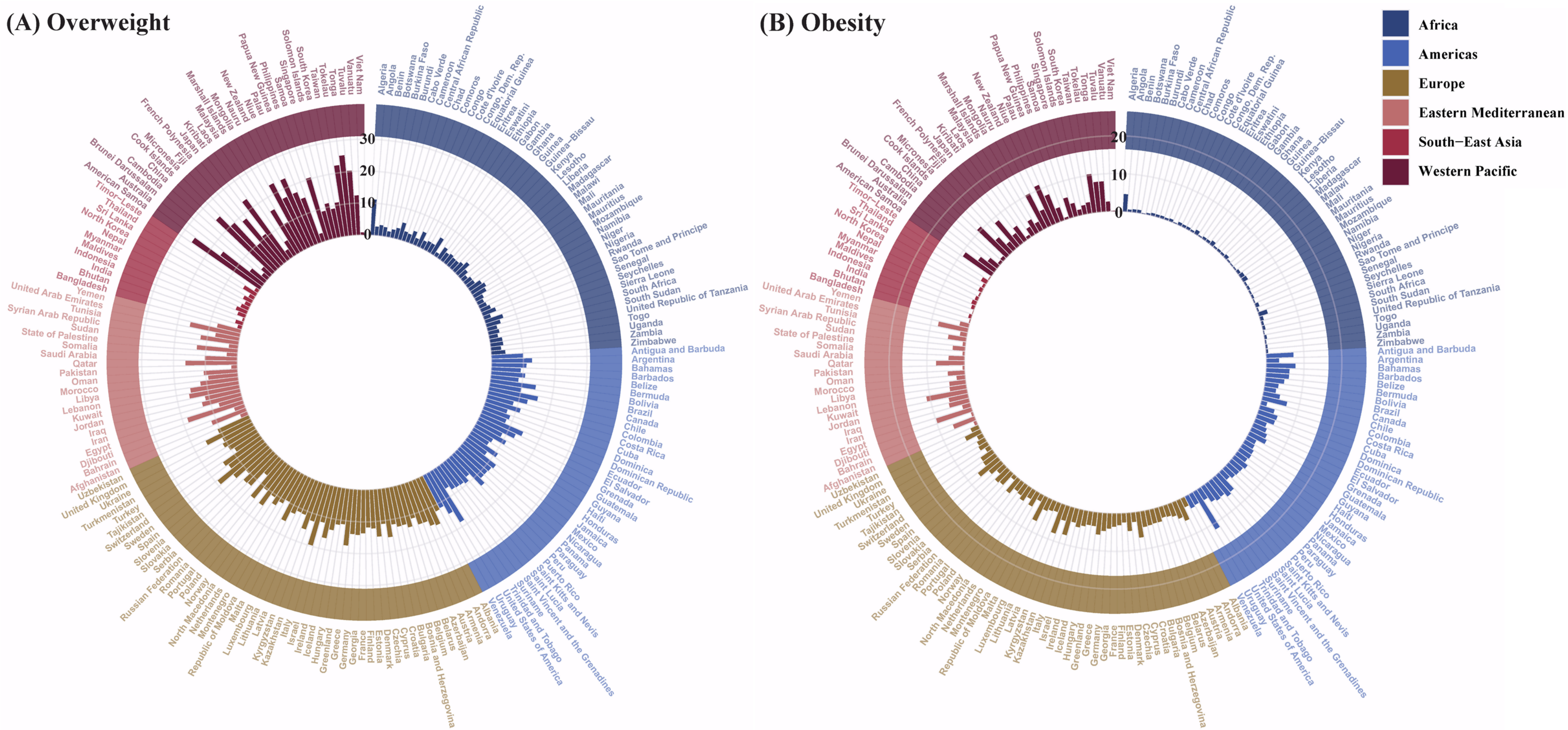

Supplementary Figure S2. Prevalence of overweight (A) and obesity (B) by country and region in 1990 among children and adolescents (age 5–19 years)

3. Supplementary Tables

Supplementary Table 1. Prevalence and number of cases of overweight, and average annual percentage changes (AAPCs) in overweight prevalence, among children and adolescents aged 5 to 19 years, by WHO region and country or territory, from 1990 to 2040.

Characteristics	1990		2022		2040		1990-2022	2023-2040
	Number of cases, thousands (95% CI)	Prevalence, % (95% CI)	Number of cases, thousands (95% CI)	Prevalence, % (95% CI)	Number of cases, thousands (95% CI)	Prevalence, % (95% CI)	AAPC, % (95% CI)	AAPC, % (95% CI)
**Africa**								
Algeria	1085(478–1691)	11.15(4.91–17.38)	2078(1033–3123)	16.7(8.3–25.09)	2723(2286–3161)	20.76(17.43–24.1)	1.28(1.25–1.3)	1.21(1.21–1.22)
Angola	113(29–196)	2.73(0.71–4.74)	1107(338–1877)	8.17(2.49–13.85)	2775(2414–3137)	12.11(10.54–13.69)	3.49(3.48–3.5)	2.2(2.18–2.22)
Benin	66(23–109)	3.32(1.17–5.46)	428(184–673)	8.41(3.61–13.21)	792(657–926)	10.69(8.87–12.5)	2.95(2.93–2.97)	1.33(1.33–1.34)
Botswana	15(4–26)	2.88(0.79–4.96)	72(25–119)	9.58(3.38–15.78)	130(116–145)	14.32(12.69–15.96)	3.84(3.81–3.87)	2.24(2.22–2.26)
Burkina Faso	61(22–100)	1.74(0.64–2.85)	496(264–728)	5.63(3–8.26)	1105(915–1295)	9.63(7.98–11.29)	3.74(3.7–3.77)	3(2.97–3.04)
Burundi	57(17–98)	2.61(0.78–4.45)	299(94–504)	5.51(1.72–9.29)	538(482–593)	7.09(6.36–7.82)	2.36(2.33–2.39)	1.41(1.4–1.42)
Cabo Verde	5(1–8)	3.37(0.98–5.77)	13(5–20)	8.74(3.43–14.05)	12(10–13)	12.35(10.79–13.91)	3.02(3–3.04)	1.93(1.92–1.94)
Cameroon	237(105–369)	5.46(2.42–8.5)	1285(651–1919)	12.56(6.37–18.75)	2449(2044–2854)	16.02(13.37–18.68)	2.64(2.6–2.67)	1.35(1.35–1.36)
Central African Republic	31(7–54)	3(0.72–5.28)	214(56–373)	9.71(2.52–16.89)	546(478–614)	14.4(12.61–16.2)	3.74(3.71–3.77)	2.21(2.19–2.22)
Chad	61(17–105)	2.66(0.73–4.59)	530(169–890)	7.24(2.31–12.17)	1352(1163–1540)	10.37(8.93–11.82)	3.19(3.16–3.22)	2.01(1.99–2.02)
Comoros	7(2–12)	4.06(1.43–6.69)	26(10–42)	9.04(3.38–14.71)	46(42–49)	12.12(11.2–13.05)	2.53(2.51–2.56)	1.64(1.63–1.65)
Congo	20(12–29)	2.23(1.28–3.18)	175(56–294)	7.84(2.52–13.16)	391(326–457)	12.34(10.28–14.41)	4(3.98–4.03)	2.54(2.51–2.56)
Cote d'Ivoire	237(100–374)	4.94(2.08–7.79)	1245(598–1892)	11.1(5.33–16.87)	2276(1910–2642)	14.25(11.96–16.55)	2.56(2.53–2.6)	1.39(1.38–1.4)
Democratic Republic of the Congo	332(106–557)	2.52(0.81–4.23)	4123(1648–6598)	10.56(4.22–16.89)	12702(10705–14699)	17.93(15.11–20.75)	4.59(4.56–4.62)	2.96(2.92–2.99)
Equatorial Guinea	5(1–8)	3.01(0.7–5.33)	58(15–101)	9.7(2.46–16.94)	129(113–145)	14.42(12.64–16.21)	3.72(3.71–3.74)	2.21(2.2–2.23)
Eritrea	22(7–37)	2.94(0.93–4.94)	95(28–162)	7.32(2.14–12.51)	177(159–194)	10.7(9.64–11.76)	2.89(2.88–2.91)	2.12(2.1–2.13)
Eswatini	20(9–32)	5.86(2.54–9.19)	49(23–74)	12.16(5.79–18.53)	63(51–75)	14.99(12.2–17.78)	2.3(2.27–2.34)	1.16(1.16–1.17)
Ethiopia	225(77–372)	1.29(0.44–2.13)	1776(867–2685)	3.92(1.92–5.93)	4920(4012–5828)	7.29(5.94–8.63)	3.56(3.52–3.59)	3.46(3.42–3.5)
Gabon	14(5–24)	4.05(1.34–6.75)	91(40–143)	11.61(5.04–18.17)	165(137–194)	14.9(12.34–17.45)	3.35(3.32–3.38)	1.39(1.38–1.4)

Gambia	11(3–19)	2.98(0.88–5.08)		82(34–129)	8.25(3.46–13.03)		160(139–181)	12.04(10.43–13.64)		3.24(3.21–3.27)	2.11(2.09–2.12)
Ghana	206(87–325)	3.47(1.47–5.48)		1156(821–1491)	10.24(7.27–13.21)		2068(1806–2330)	14.98(13.08–16.87)		3.45(3.42–3.47)	2.11(2.09–2.12)
Guinea	70(24–117)	3.05(1.04–5.07)		467(209–724)	8.98(4.02–13.94)		1012(874–1150)	13.84(11.96–15.73)		3.44(3.42–3.46)	2.41(2.39–2.43)
Guinea-Bissau	11(3–19)	3.01(0.93–5.09)		65(21–109)	8.39(2.67–14.12)		124(108–140)	12.27(10.68–13.87)		3.26(3.25–3.28)	2.12(2.1–2.14)
Kenya	345(134–555)	3.64(1.42–5.86)		1499(933–2065)	7.56(4.7–10.41)		1890(1437–2344)	7.88(5.99–9.77)		2.32(2.3–2.34)	0.23(0.23–0.23)
Lesotho	33(14–52)	4.96(2.15–7.76)		66(29–102)	8.52(3.81–13.23)		78(60–97)	9.76(7.48–12.04)		1.69(1.65–1.73)	0.76(0.75–0.76)
Liberia	25(9–41)	3.03(1.05–5.01)		160(85–235)	7.94(4.2–11.69)		314(273–354)	11.62(10.12–13.12)		3.07(3.05–3.09)	2.11(2.1–2.13)
Madagascar	86(22–149)	1.83(0.47–3.18)		553(179–927)	5.08(1.65–8.51)		1289(1101–1477)	8.23(7.03–9.44)		3.25(3.23–3.27)	2.7(2.67–2.73)
Malawi	94(37–150)	2.66(1.06–4.27)		540(259–820)	6.75(3.24–10.25)		1168(999–1338)	10.66(9.11–12.21)		2.95(2.93–2.97)	2.55(2.53–2.58)
Mali	96(33–159)	2.71(0.93–4.49)		979(427–1530)	10.54(4.6–16.47)		2454(2074–2834)	16.21(13.7–18.73)		4.36(4.29–4.42)	2.4(2.38–2.42)
Mauritania	33(11–55)	4.41(1.51–7.3)		249(129–368)	13.45(6.99–19.9)		574(507–641)	19.99(17.66–22.32)		3.55(3.54–3.57)	2.21(2.19–2.23)
Mauritius	17(6–27)	5.13(1.79–8.47)		40(26–53)	17.28(11.32–23.23)		36(28–44)	22.38(17.49–27.27)		3.87(3.84–3.9)	1.44(1.43–1.45)
Mozambique	220(95–345)	4.4(1.9–6.91)		1037(514–1561)	8.19(4.06–12.33)		1918(1635–2200)	9.4(8.01–10.79)		1.95(1.93–1.97)	0.77(0.76–0.77)
Namibia	14(4–23)	2.69(0.83–4.55)		88(29–146)	9.3(3.11–15.49)		169(148–190)	13.84(12.14–15.55)		3.96(3.94–3.99)	2.21(2.2–2.23)
Niger	65(18–112)	2.1(0.58–3.61)		684(281–1088)	6.67(2.74–10.61)		1724(1468–1981)	9.71(8.26–11.15)		3.7(3.65–3.74)	2.08(2.07–2.1)
Nigeria	893(354–1431)	2.44(0.97–3.91)		4907(2314–7500)	5.78(2.73–8.84)		8751(7325–10178)	7.7(6.45–8.96)		2.73(2.72–2.75)	1.6(1.59–1.61)
Rwanda	95(40–150)	3.22(1.36–5.07)		395(232–558)	8.06(4.74–11.38)		778(666–890)	12.1(10.36–13.84)		2.93(2.89–2.97)	2.26(2.24–2.28)
Sao Tome and Principe	2(1–3)	4.46(1.52–7.39)		9(4–14)	10.8(4.58–17.03)		14(11–17)	13.13(10.61–15.65)		2.81(2.76–2.86)	1.09(1.08–1.09)
Senegal	68(22–114)	2.22(0.71–3.72)		383(128–638)	5.94(1.98–9.9)		802(680–924)	9.31(7.89–10.73)		3.13(3.11–3.15)	2.51(2.49–2.53)
Seychelles	1(1–2)	5.84(3.24–8.43)		4(3–4)	15.88(13.27–18.49)		6(5–7)	21.46(17.55–25.37)		3.17(3.13–3.21)	1.67(1.66–1.68)
Sierra Leone	68(25–111)	4.55(1.67–7.43)		289(161–417)	9.71(5.42–14)		477(372–582)	12.26(9.57–14.96)		2.38(2.34–2.42)	1.3(1.3–1.31)
South Africa	420(214–626)	3.09(1.57–4.61)		2194(1603–2784)	13.98(10.22–17.74)		2618(814–4422)	14.54(4.52–24.56)		4.86(4.76–4.97)	0.22(0.21–0.23)
South Sudan	64(18–110)	3.45(0.99–5.92)		346(102–591)	7.8(2.29–13.31)		580(539–621)	10.68(9.93–11.44)		2.58(2.56–2.59)	1.76(1.75–1.77)
Togo	60(20–100)	3.9(1.32–6.48)		286(130–442)	8.74(3.97–13.52)		458(362–554)	10.08(7.96–12.2)		2.56(2.51–2.6)	0.79(0.79–0.8)
Uganda	274(126–422)	3.97(1.83–6.12)		1389(777–2000)	7.41(4.15–10.67)		2799(2373–3225)	10.03(8.5–11.55)		1.96(1.92–1.99)	1.69(1.68–1.7)
United Republic of Tanzania	282(104–460)	2.73(1.01–4.46)		1480(672–2287)	6.09(2.77–9.42)		3265(2975–3554)	8.38(7.64–9.12)		2.54(2.52–2.55)	1.78(1.77–1.79)
Zambia	100(36–165)	3.16(1.13–5.19)		655(275–1036)	8.52(3.57–13.46)		1417(1261–1573)	12.58(11.19–13.96)		3.15(3.14–3.16)	2.17(2.16–2.19)
Zimbabwe	186(86–287)	4.39(2.01–6.77)		494(228–761)	7.96(3.67–12.24)		808(662–954)	10.32(8.45–12.19)		1.88(1.82–1.93)	1.45(1.44–1.46)
**Americas**											
Antigua and Barbuda	2(1–3)	9.9(4.13–15.67)		4(2–6)	20.14(9.2–31.08)		4(4–4)	25.78(24.29–27.26)		2.25(2.22–2.28)	1.37(1.36–1.38)

Argentina	1189(664–1713)	12.66(7.08–18.25)		2267(1748–2785)	20.85(16.08–25.62)		1490(1135–1846)	19.58(14.91–24.24)		1.56(1.52–1.6)	-0.35(-0.35–-0.35)
Bahamas	9(4–14)	10.94(4.78–17.11)		19(11–26)	23.07(13.98–32.16)		20(18–21)	29.79(27.44–32.13)		2.36(2.33–2.39)	1.42(1.41–1.43)
Barbados	6(3–10)	9.19(3.93–14.46)		10(5–15)	19.05(9.48–28.62)		11(11–11)	24.64(23.96–25.32)		2.3(2.29–2.32)	1.43(1.42–1.44)
Belize	8(3–12)	10.65(4.53–16.78)		25(13–38)	22.03(10.99–33.07)		30(28–32)	27.39(25.44–29.34)		2.3(2.28–2.31)	1.2(1.19–1.21)
Bermuda	1(0–2)	8.71(3.08–14.33)		2(1–3)	18.56(7.13–29.99)		2(2–2)	24.41(23.58–25.25)		2.39(2.37–2.41)	1.52(1.51–1.53)
Bolivia (Plurinational State of)	364(192–536)	14.36(7.58–21.15)		863(532–1194)	24.03(14.82–33.24)		1179(1073–1285)	30.15(27.43–32.86)		1.62(1.6–1.64)	1.26(1.25–1.26)
Brazil	4977(4030–5925)	9.97(8.07–11.86)		9002(7702–10302)	20.4(17.45–23.34)		9432(8401–10462)	25.71(22.9–28.52)		2.27(2.25–2.28)	1.29(1.28–1.29)
Canada	899(707–1090)	15.59(12.27–18.92)		1063(807–1319)	17.04(12.94–21.14)		965(793–1137)	15.36(12.62–18.09)		0.27(0.25–0.3)	-0.58(-0.58–-0.57)
Chile	487(301–674)	12.67(7.82–17.52)		1114(790–1437)	30.41(21.58–39.23)		1041(965–1117)	38.87(36.03–41.7)		2.77(2.74–2.8)	1.36(1.35–1.37)
Colombia	928(537–1320)	8.48(4.91–12.06)		1940(1454–2427)	17.04(12.76–21.31)		2264(1918–2610)	21.73(18.41–25.05)		2.2(2.18–2.22)	1.35(1.34–1.36)
Costa Rica	112(57–166)	11.22(5.71–16.72)		215(142–287)	20.11(13.34–26.88)		179(158–201)	23.77(20.94–26.6)		1.84(1.82–1.85)	0.93(0.92–0.93)
Cuba	193(100–287)	7.38(3.83–10.93)		365(262–467)	20.07(14.43–25.7)		395(342–449)	30.66(26.51–34.8)		3.18(3.16–3.2)	2.37(2.35–2.38)
Dominica	2(1–3)	8.77(3.8–13.75)		2(1–4)	18.25(8.55–27.95)		2(2–3)	23.56(22.47–24.66)		2.32(2.3–2.33)	1.43(1.42–1.43)
Dominican Republic	210(102–318)	8.08(3.93–12.24)		521(283–759)	17.28(9.38–25.19)		691(639–743)	23.87(22.06–25.67)		2.4(2.4–2.41)	1.8(1.79–1.81)
Ecuador	543(302–783)	14.45(8.04–20.86)		980(746–1215)	20.92(15.92–25.92)		983(858–1108)	24.22(21.14–27.3)		1.16(1.14–1.17)	0.81(0.81–0.82)
El Salvador	255(135–374)	12.6(6.69–18.52)		296(177–415)	17.63(10.56–24.71)		248(192–304)	17.91(13.87–21.95)		1.05(1.03–1.08)	0.09(0.09–0.09)
Grenada	3(1–4)	8.73(3.09–14.37)		5(2–8)	18.68(7.36–30)		5(4–5)	24.92(23.27–26.57)		2.41(2.39–2.42)	1.61(1.6–1.62)
Guatemala	380(218–541)	10.94(6.29–15.59)		876(707–1045)	15.06(12.15–17.96)		791(579–1003)	13.52(9.9–17.14)		0.99(0.96–1.03)	-0.59(-0.6–-0.59)
Guyana	19(8–29)	7.03(2.99–11.06)		33(15–51)	14.33(6.65–22.01)		45(41–49)	19.25(17.69–20.81)		2.25(2.23–2.27)	1.65(1.64–1.66)
Haiti	160(61–258)	6.43(2.47–10.4)		513(222–803)	14.31(6.19–22.44)		741(686–795)	19.75(18.3–21.2)		2.53(2.52–2.55)	1.8(1.78–1.81)
Honduras	213(96–331)	10.79(4.85–16.73)		611(282–940)	18.99(8.77–29.2)		796(700–892)	22.18(19.5–24.86)		1.78(1.76–1.81)	0.86(0.86–0.87)
Jamaica	62(31–94)	7.69(3.77–11.61)		110(59–161)	17.75(9.54–25.97)		100(91–110)	23.87(21.54–26.2)		2.65(2.64–2.66)	1.64(1.63–1.65)
Mexico	3800(2729–4871)	12.36(8.87–15.84)		7088(6260–7915)	21.24(18.76–23.72)		6370(4484–8256)	21.13(14.87–27.38)		1.7(1.64–1.76)	-0.03(-0.03–-0.03)
Nicaragua	200(102–299)	11.76(5.98–17.54)		401(196–606)	20.3(9.92–30.68)		466(404–529)	23.49(20.36–26.62)		1.72(1.7–1.74)	0.81(0.81–0.81)
Panama	97(56–137)	11.51(6.65–16.37)		262(204–320)	23.3(18.12–28.47)		334(308–361)	30.19(27.81–32.56)		2.23(2.21–2.25)	1.44(1.43–1.45)
Paraguay	165(78–253)	11.51(5.41–17.61)		372(235–509)	20.05(12.65–27.45)		462(410–513)	23.07(20.5–25.64)		1.75(1.72–1.79)	0.78(0.77–0.78)
Peru	1063(626–1500)	13.35(7.86–18.84)		2043(1808–2278)	24.16(21.38–26.93)		2822(2389–3255)	34.87(29.52–40.22)		1.87(1.84–1.89)	2.04(2.03–2.06)

Puerto Rico	79(28–130)	8.07(2.84–13.29)		83(32–134)	16.87(6.45–27.29)		58(55–61)	22.34(21.17–23.52)		2.33(2.31–2.35)	1.56(1.55–1.57)
Saint Kitts and Nevis	1(1–2)	9.48(4.11–14.85)		2(1–3)	19.34(9.48–29.2)		2(2–2)	24.52(23.25–25.79)		2.25(2.22–2.28)	1.31(1.3–1.31)
Saint Lucia	4(2–7)	8.82(3.34–14.31)		6(3–9)	17.65(8.58–26.73)		6(6–7)	22.54(21.85–23.23)		2.19(2.18–2.2)	1.35(1.34–1.36)
Saint Vincent and the Grenadines	4(1–6)	8.65(3.51–13.79)		4(2–5)	17.08(10.51–23.64)		4(3–4)	21.82(20.73–22.91)		2.14(2.13–2.15)	1.37(1.37–1.38)
Suriname	12(4–19)	8.4(2.94–13.86)		29(11–47)	17.77(6.9–28.63)		38(37–39)	23.26(22.43–24.09)		2.37(2.36–2.38)	1.5(1.49–1.51)
Trinidad and Tobago	28(15–41)	7.08(3.8–10.36)		50(24–77)	17.86(8.5–27.21)		55(52–59)	24.74(23.12–26.36)		2.94(2.93–2.96)	1.81(1.8–1.82)
United States of America	9710(8564–10856)	18.42(16.24–20.59)		13819(11046–16592)	21.36(17.07–25.64)		13259(11658–14861)	22.46(19.74–25.17)		0.47(0.45–0.48)	0.28(0.28–0.28)
Uruguay	113(66–159)	14.37(8.41–20.32)		156(120–192)	22.3(17.15–27.44)		114(91–137)	23.09(18.43–27.76)		1.38(1.33–1.43)	0.19(0.19–0.2)
Venezuela (Bolivarian Republic of)	789(360–1219)	11.46(5.22–17.69)		1703(952–2455)	21.2(11.85–30.55)		1628(1440–1817)	24.9(22.02–27.77)		1.94(1.92–1.96)	0.89(0.88–0.89)
**Europe**											
Albania	118(68–169)	11.82(6.8–16.83)		77(54–100)	14.98(10.57–19.39)		47(36–59)	14.42(11–17.85)		0.73(0.7–0.76)	-0.21(-0.21–-0.21)
Andorra	2(1–3)	15.8(7.6–24)		2(1–3)	17.34(6.66–28.03)		1(1–2)	15.3(11.8–18.81)		0.3(0.27–0.33)	-0.7(-0.7–-0.69)
Armenia	147(85–209)	15.26(8.8–21.72)		87(57–117)	15.33(10.05–20.61)		77(65–90)	18.21(15.32–21.1)		0.02(-0.02–0.06)	0.96(0.95–0.96)
Austria	192(151–232)	13.56(10.69–16.43)		234(194–274)	17.9(14.86–20.95)		199(163–234)	17.5(14.37–20.63)		0.87(0.85–0.9)	-0.13(-0.13–-0.12)
Azerbaijan	295(146–445)	13.55(6.69–20.4)		383(179–588)	16.23(7.58–24.88)		363(326–401)	19.27(17.3–21.25)		0.57(0.55–0.59)	0.96(0.95–0.96)
Belarus	253(105–400)	11.11(4.62–17.61)		224(104–343)	14.22(6.61–21.83)		142(118–167)	15.62(12.92–18.31)		0.77(0.75–0.8)	0.52(0.52–0.52)
Belgium	252(165–340)	13.52(8.82–18.23)		295(209–381)	14.85(10.53–19.16)		254(212–295)	15.77(13.19–18.36)		0.28(0.26–0.31)	0.34(0.34–0.34)
Bosnia and Herzegovina	137(67–207)	12.36(6.07–18.66)		91(51–130)	19.56(11.04–28.09)		73(56–90)	21.88(16.83–26.93)		1.46(1.39–1.52)	0.62(0.62–0.62)
Bulgaria	235(190–280)	12.53(10.12–14.95)		173(133–213)	17.15(13.21–21.09)		164(139–189)	19.45(16.51–22.39)		1(0.97–1.02)	0.7(0.69–0.7)
Croatia	115(71–158)	11.54(7.17–15.9)		103(77–128)	17.53(13.16–21.91)		83(63–103)	18.3(13.9–22.7)		1.32(1.27–1.36)	0.23(0.23–0.24)
Cyprus	29(17–41)	15.32(9.17–21.48)		39(28–50)	18.88(13.39–24.37)		44(37–51)	20.04(16.81–23.27)		0.65(0.63–0.67)	0.33(0.33–0.33)
Czechia	263(168–357)	10.94(7.01–14.86)		181(126–236)	10.83(7.53–14.12)		86(47–125)	6.59(3.6–9.59)		-0.02(-0.07–0.03)	-2.73(-2.76–-2.7)
Denmark	141(120–161)	14.76(12.59–16.94)		132(100–164)	13.45(10.21–16.7)		81(36–125)	8.68(3.86–13.5)		-0.28(-0.34–-0.23)	-2.42(-2.44–-2.39)
Estonia	41(23–59)	12.11(6.66–17.56)		39(32–46)	17.75(14.43–21.07)		32(27–37)	21.59(18.33–24.85)		1.2(1.19–1.22)	1.08(1.08–1.09)

Finland	112(87–137)	11.75(9.14–14.36)		165(132–198)	18.11(14.48–21.73)		142(124–159)	20.62(18.04–23.19)		1.36(1.34–1.38)	0.72(0.72–0.72)
France	1630(1056–2204)	13.6(8.81–18.38)		1498(1020–1977)	12.54(8.54–16.54)		1082(767–1397)	10.69(7.58–13.81)		-0.26(-0.29–-0.23)	-0.88(-0.88–-0.88)
Georgia	185(91–279)	14.13(6.95–21.31)		128(84–172)	16.74(11.02–22.46)		130(117–143)	20.38(18.37–22.39)		0.53(0.52–0.55)	1.09(1.09–1.1)
Germany	1795(1326–2265)	13.94(10.29–17.59)		1952(1369–2535)	16.88(11.84–21.92)		1723(1430–2017)	16.32(13.54–19.1)		0.59(0.56–0.62)	-0.19(-0.19–-0.19)
Greece	391(293–489)	17.64(13.21–22.07)		349(285–412)	22.47(18.4–26.55)		215(164–265)	20.47(15.67–25.26)		0.76(0.73–0.78)	-0.52(-0.52–-0.52)
Greenland	1(1–2)	11.14(5.07–17.21)		1(0–1)	8.43(3.24–13.63)		1(0–1)	5.56(2.05–9.07)		-0.88(-0.96–-0.81)	-2.29(-2.31–-2.27)
Hungary	261(153–369)	11.5(6.73–16.26)		252(205–298)	17.55(14.32–20.79)		241(196–286)	19.01(15.48–22.55)		1.34(1.31–1.37)	0.44(0.44–0.44)
Iceland	9(4–13)	13.5(6.21–20.79)		11(4–18)	15.59(5.8–25.38)		11(9–12)	15.29(12.75–17.82)		0.45(0.44–0.47)	-0.11(-0.11–-0.11)
Ireland	164(108–221)	16.4(10.8–22.01)		179(142–216)	17.35(13.78–20.92)		112(79–145)	12.67(8.98–16.36)		0.18(0.14–0.21)	-1.74(-1.75–-1.72)
Israel	160(129–191)	11.78(9.5–14.05)		330(244–415)	13.89(10.27–17.5)		421(380–463)	14.88(13.42–16.34)		0.52(0.51–0.53)	0.39(0.38–0.39)
Italy	2159(1791–2526)	19.68(16.33–23.03)		1439(1079–1799)	17.66(13.24–22.08)		648(392–905)	11.23(6.79–15.68)		-0.32(-0.36–-0.28)	-2.49(-2.52–-2.47)
Kazakhstan	613(350–876)	12.31(7.03–17.6)		770(566–975)	14.74(10.82–18.66)		1288(1104–1471)	20.61(17.68–23.55)		0.57(0.54–0.6)	1.87(1.85–1.88)
Kyrgyzstan	225(164–286)	15.5(11.3–19.7)		192(104–279)	9.36(5.09–13.63)		203(136–270)	8.7(5.82–11.59)		-1.57(-1.59–-1.55)	-0.39(-0.4–-0.39)
Latvia	61(32–91)	11.23(5.84–16.63)		46(31–61)	15.12(10.26–19.98)		34(26–42)	17.07(13.1–21.03)		0.94(0.89–0.99)	0.67(0.67–0.68)
Lithuania	91(48–134)	11.06(5.82–16.3)		57(38–76)	13.43(8.94–17.92)		44(36–51)	14.87(12.36–17.37)		0.61(0.59–0.63)	0.56(0.55–0.56)
Luxembourg	8(4–12)	12.28(5.55–19)		15(5–24)	14.11(5.12–23.09)		16(14–19)	14.62(12.62–16.62)		0.43(0.41–0.45)	0.2(0.2–0.2)
Malta	13(8–19)	15.6(9.12–22.08)		11(8–15)	16.11(10.96–21.27)		9(8–11)	15.43(12.58–18.27)		0.1(0.06–0.13)	-0.24(-0.24–-0.24)
Montenegro	18(12–24)	11.12(7.31–14.94)		21(17–24)	17.98(14.88–21.08)		17(14–21)	18.79(14.8–22.79)		1.51(1.49–1.53)	0.24(0.24–0.25)
Netherlands (Kingdom of the)	370(234–506)	12.8(8.08–17.51)		372(226–518)	12.88(7.82–17.95)		356(274–438)	12.68(9.76–15.59)		0.01(-0.02–0.04)	-0.09(-0.09–-0.09)
North Macedonia	60(32–88)	10.8(5.82–15.77)		52(34–69)	16.05(10.66–21.44)		42(35–48)	18.65(15.81–21.5)		1.25(1.23–1.27)	0.84(0.83–0.84)
Norway	127(88–165)	15.12(10.52–19.72)		163(119–207)	16.99(12.39–21.59)		148(117–178)	17.87(14.23–21.52)		0.35(0.33–0.38)	0.28(0.28–0.28)
Poland	993(775–1210)	10.48(8.18–12.77)		944(745–1144)	15.97(12.59–19.35)		629(459–800)	15.38(11.21–19.56)		1.32(1.28–1.37)	-0.21(-0.22–-0.21)
Portugal	346(276–415)	14.94(11.92–17.96)		199(147–250)	13.66(10.09–17.23)		78(18–138)	5.99(1.35–10.62)		-0.28(-0.32–-0.24)	-4.48(-4.57–-4.39)

Republic of Moldova	111(53–168)	9.68(4.68–14.68)		80(43–117)	13.36(7.24–19.48)		71(62–80)	17.38(15.12–19.64)		1.02(0.99–1.04)	1.46(1.46–1.47)
Romania	373(234–512)	6.79(4.26–9.33)		448(358–537)	14.19(11.34–17.03)		405(259–551)	15.94(10.19–21.7)		2.35(2.3–2.41)	0.64(0.64–0.64)
Russian Federation	4124(3307–4941)	12.57(10.08–15.06)		3468(2571–4365)	13.47(9.99–16.96)		2663(1622–3704)	13.83(8.42–19.24)		0.2(0.07–0.33)	0.15(0.14–0.15)
Serbia	203(150–255)	12.86(9.52–16.2)		197(151–244)	19.72(15.11–24.33)		191(163–218)	23.28(19.91–26.65)		1.34(1.33–1.35)	0.92(0.92–0.93)
Slovakia	139(101–177)	10.28(7.47–13.09)		141(101–181)	16.49(11.79–21.19)		133(109–157)	18.98(15.5–22.47)		1.5(1.47–1.53)	0.78(0.77–0.78)
Slovenia	62(58–66)	14.16(13.2–15.13)		59(53–64)	18.4(16.68–20.13)		43(32–53)	16.25(12.28–20.23)		0.83(0.8–0.86)	-0.69(-0.7–-0.69)
Spain	1712(1272–2153)	19.13(14.21–24.05)		1465(1205–1724)	20.42(16.8–24.04)		915(758–1072)	18.21(15.09–21.33)		0.2(0.19–0.22)	-0.64(-0.64–-0.63)
Sweden	207(186–229)	13.41(12.03–14.79)		307(232–383)	16.62(12.55–20.7)		288(233–344)	18.41(14.85–21.97)		0.68(0.64–0.73)	0.57(0.57–0.57)
Switzerland	170(114–226)	14.35(9.59–19.1)		202(168–235)	15.36(12.83–17.89)		159(126–192)	12.63(10.02–15.24)		0.21(0.18–0.24)	-1.08(-1.09–-1.08)
Tajikistan	170(78–262)	8.89(4.06–13.72)		248(147–349)	7.62(4.52–10.72)		321(272–371)	7.84(6.64–9.04)		-0.48(-0.49–-0.46)	0.16(0.16–0.16)
Turkey	1966(1065–2868)	10.28(5.57–14.99)		4127(2937–5317)	20.98(14.93–27.03)		4672(3987–5357)	31.2(26.62–35.77)		2.26(2.22–2.31)	2.21(2.19–2.23)
Turkmenistan	149(69–228)	11.28(5.28–17.29)		244(138–350)	12.34(6.98–17.7)		353(318–389)	15.67(14.1–17.23)		0.28(0.26–0.3)	1.33(1.33–1.34)
Ukraine	1153(618–1688)	10.53(5.64–15.41)		822(621–1024)	12.6(9.51–15.69)		373(197–548)	10.37(5.49–15.25)		0.57(0.52–0.63)	-1.08(-1.09–-1.08)
United Kingdom	1751(1547–1955)	15.63(13.8–17.45)		2341(1992–2691)	19.13(16.27–21.99)		1903(1443–2362)	16.82(12.76–20.88)		0.64(0.59–0.68)	-0.71(-0.72–-0.71)
Uzbekistan	759(384–1134)	10.69(5.41–15.97)		1233(536–1930)	13.52(5.88–21.16)		2424(2119–2730)	17.63(15.41–19.84)		0.75(0.72–0.77)	1.47(1.47–1.48)
**Eastern Mediterranean**											
Afghanistan	109(31–186)	2.34(0.67–4.01)		2123(1105–3142)	13.5(7.02–19.98)		5795(4843–6747)	23.89(19.96–27.81)		5.64(5.61–5.67)	3.18(3.14–3.22)
Bahrain	16(9–23)	11.49(6.52–16.45)		58(38–78)	20.39(13.33–27.45)		87(71–103)	27.26(22.4–32.12)		1.82(1.77–1.87)	1.61(1.6–1.62)
Djibouti	7(2–13)	3.34(0.88–5.8)		27(7–46)	7.71(2.08–13.33)		40(36–43)	10.77(9.76–11.78)		2.65(2.64–2.66)	1.87(1.85–1.88)
Egypt	3793(2461–5125)	18.32(11.89–24.75)		7760(5346–10175)	22(15.16–28.85)		7803(6068–9538)	20.31(15.79–24.82)		0.57(0.56–0.59)	-0.44(-0.45–-0.44)
Iran (Islamic Republic of)	1616(1216–2015)	7.53(5.67–9.39)		3617(2861–4374)	17.8(14.08–21.52)		4251(3191–5311)	25.64(19.24–32.03)		2.74(2.69–2.78)	2.03(2.02–2.04)
Iraq	871(413–1329)	12.56(5.96–19.16)		3205(1755–4655)	20.56(11.26–29.86)		5001(4311–5690)	26.36(22.73–30)		1.55(1.53–1.57)	1.39(1.38–1.39)
Jordan	160(86–234)	11.21(6.04–16.39)		778(563–993)	22.27(16.11–28.42)		1219(1033–1405)	32.94(27.9–37.97)		2.18(2.12–2.23)	2.18(2.16–2.2)
Kuwait	89(63–115)	15.98(11.24–20.71)		222(155–289)	25.8(18.05–33.55)		337(280–395)	34.39(28.58–40.2)		1.51(1.43–1.59)	1.6(1.59–1.61)

Lebanon	186(121–251)	15.32(9.96–20.68)		376(209–543)	23.24(12.91–33.57)		403(352–455)	28.07(24.51–31.64)		1.31(1.29–1.34)	1.05(1.05–1.06)
Libya	210(111–310)	11.92(6.26–17.57)		402(192–611)	19.51(9.34–29.69)		493(421–566)	25.15(21.44–28.85)		1.56(1.54–1.58)	1.41(1.4–1.42)
Morocco	844(414–1274)	9.26(4.54–13.97)		1477(1015–1938)	15.19(10.44–19.94)		1898(1618–2179)	20.57(17.53–23.61)		1.56(1.54–1.59)	1.69(1.68–1.7)
State of Palestine	110(62–158)	12.83(7.23–18.42)		330(210–450)	17.64(11.24–24.04)		464(388–540)	20.14(16.84–23.45)		1(0.98–1.03)	0.74(0.74–0.74)
Oman	68(33–103)	10.58(5.08–16.08)		211(128–294)	19.72(11.94–27.51)		416(352–479)	28.11(23.83–32.39)		1.98(1.95–2.02)	1.97(1.96–1.98)
Pakistan	765(464–1066)	1.82(1.1–2.53)		8809(5916–11702)	10.39(6.98–13.8)		20072(16610–23534)	19.13(15.83–22.43)		5.61(5.59–5.62)	3.41(3.36–3.45)
Qatar	17(9–25)	16.24(8.67–23.81)		87(46–129)	22.67(11.96–33.37)		137(111–164)	24.8(19.99–29.62)		1.05(1.03–1.07)	0.5(0.5–0.5)
Saudi Arabia	369(280–457)	10.15(7.72–12.59)		1756(1124–2388)	23.18(14.84–31.53)		3194(2444–3945)	35.01(26.78–43.24)		2.63(2.57–2.69)	2.3(2.28–2.32)
Somalia	87(24–151)	3.31(0.9–5.71)		537(142–932)	7.73(2.04–13.42)		1339(1207–1470)	10.82(9.76–11.88)		2.69(2.66–2.72)	1.88(1.86–1.89)
Sudan	242(67–417)	2.86(0.79–4.93)		1422(522–2322)	7.9(2.9–12.91)		2973(2680–3267)	11.52(10.38–12.66)		3.23(3.21–3.24)	2.1(2.09–2.12)
Syrian Arab Republic	609(256–962)	12.03(5.05–19.01)		1542(638–2447)	19.9(8.23–31.57)		2612(2239–2985)	26.33(22.57–30.09)		1.59(1.56–1.61)	1.56(1.55–1.57)
Tunisia	221(134–307)	7.56(4.6–10.52)		484(242–726)	16.7(8.35–25.06)		560(455–666)	24.2(19.65–28.76)		2.51(2.48–2.54)	2.07(2.05–2.08)
United Arab Emirates	80(46–115)	16.02(9.17–22.87)		385(258–513)	23.73(15.87–31.59)		575(500–650)	27.76(24.13–31.38)		1.24(1.22–1.26)	0.87(0.87–0.88)
Yemen	426(221–631)	7.61(3.95–11.27)		1416(604–2228)	10.38(4.43–16.33)		3145(2620–3671)	14.19(11.82–16.56)		0.99(0.96–1.02)	1.74(1.73–1.75)
**South-East Asia**											
Bangladesh	570(156–983)	1.36(0.37–2.35)		3157(1963–4350)	6.37(3.96–8.77)		5279(4553–6005)	10.46(9.02–11.9)		4.94(4.91–4.97)	2.77(2.74–2.79)
Bhutan	5(1–9)	2.45(0.62–4.28)		24(12–35)	12.23(6.21–18.26)		30(26–35)	20.6(17.52–23.68)		5.16(5.13–5.18)	2.91(2.88–2.94)
Democratic People's Republic of Korea	178(50–307)	3.06(0.85–5.26)		589(187–990)	11.82(3.76–19.87)		866(776–956)	18.35(16.44–20.26)		4.32(4.3–4.33)	2.46(2.44–2.48)
India	1786(1207–2365)	0.6(0.41–0.8)		25874(19756–31992)	6.86(5.24–8.49)		41102(30544–51660)	12.04(8.95–15.13)		7.94(7.83–8.04)	3.14(3.1–3.17)
Indonesia	1658(1032–2284)	2.62(1.63–3.6)		9087(6304–11870)	12.84(8.91–16.77)		15646(12835–18456)	23.34(19.15–27.53)		5.12(5.07–5.16)	3.33(3.29–3.37)
Maldives	3(1–5)	3.69(1.33–6.05)		20(13–27)	18.85(12.23–25.47)		27(22–32)	33.86(27.38–40.34)		5.23(5.2–5.26)	3.25(3.22–3.29)
Myanmar	358(119–597)	2.62(0.87–4.37)		1227(476–1977)	9.22(3.58–14.85)		1804(1625–1984)	14.07(12.67–15.47)		4.01(3.99–4.02)	2.36(2.34–2.38)
Nepal	130(41–219)	1.87(0.59–3.15)		607(312–901)	6.84(3.52–10.17)		923(764–1082)	11.61(9.61–13.61)		4.13(4.1–4.16)	2.95(2.92–2.98)
Sri Lanka	97(31–163)	1.8(0.57–3.03)		506(269–744)	9.51(5.05–13.96)		761(649–874)	15.92(13.58–18.27)		5.34(5.32–5.36)	2.88(2.85–2.91)
Thailand	781(550–1013)	4.57(3.22–5.93)		1658(1320–1995)	13.46(10.72–16.2)		1586(1420–1752)	18.39(16.47–20.32)		3.42(3.39–3.45)	1.74(1.73–1.75)
Timor-Leste	3(1–5)	1.24(0.36–2.11)		14(6–21)	2.85(1.24–4.46)		17(15–20)	3.54(3.04–4.05)		2.63(2.59–2.66)	1.23(1.22–1.23)
**Western Pacific**											
American Samoa	4(3–5)	26.01(17.55–34.48)		4(2–6)	26.2(12.5–39.9)		2(2–2)	25.08(19.89–30.27)		0.03(0–0.06)	-0.24(-0.24–-0.24)

Australia	636(539–734)	16.27(13.78–18.77)		988(786–1190)	20.62(16.41–24.83)		975(803–1147)	19.83(16.33–23.33)		0.74(0.71–0.77)	-0.22(-0.22–-0.22)
Brunei Darussalam	5(2–7)	5.9(2.56–9.25)		19(14–25)	19.61(13.89–25.33)		24(21–27)	26.47(22.9–30.04)		3.82(3.79–3.84)	1.67(1.66–1.68)
Cambodia	55(17–93)	2.2(0.67–3.73)		428(191–665)	8.58(3.82–13.34)		708(596–820)	13.14(11.07–15.22)		4.34(4.29–4.38)	2.37(2.35–2.39)
China	11569(9731–13408)	3.52(2.96–4.08)		28212(21146–35278)	10.91(8.18–13.64)		25068(21362–28773)	20.09(17.12–23.06)		3.57(3.5–3.65)	2.84(2.81–2.87)
Cook Islands	1(1–2)	24.46(14.6–34.31)		1(1–1)	24.88(15.03–34.73)		0(0–0)	21.56(17.36–25.77)		0.04(0–0.08)	-0.79(-0.8–-0.79)
Fiji	26(13–39)	9.69(4.73–14.64)		46(28–64)	18.46(11.37–25.56)		67(57–78)	28.25(24–32.49)		2.05(2.01–2.09)	2.37(2.35–2.39)
French Polynesia	12(6–18)	18.38(9.67–27.09)		13(8–19)	21.12(12.55–29.69)		9(8–10)	22.57(19.91–25.24)		0.44(0.42–0.45)	0.37(0.37–0.37)
Japan	2836(2589–3084)	10.84(9.9–11.79)		1929(1582–2277)	11.88(9.74–14.02)		1708(1501–1915)	14.79(12.99–16.59)		0.27(0.25–0.29)	1.22(1.22–1.22)
Kiribati	6(4–8)	23.2(14.13–32.27)		10(5–15)	24.79(12.93–36.66)		11(8–13)	21.9(17.17–26.62)		0.21(0.18–0.24)	-0.69(-0.69–-0.68)
Lao People's Democratic Republic	45(14–76)	2.8(0.86–4.73)		227(76–379)	10.04(3.36–16.73)		375(333–417)	15.7(13.94–17.45)		4.08(4.06–4.1)	2.49(2.47–2.51)
Malaysia	320(187–453)	5.29(3.1–7.48)		1366(1021–1711)	16.15(12.06–20.23)		1411(1146–1676)	19.55(15.88–23.22)		3.54(3.51–3.57)	1.07(1.06–1.07)
Marshall Islands	3(2–4)	15.73(8.53–22.93)		3(1–4)	18.31(9.5–27.13)		1(1–2)	17.88(14.96–20.8)		0.49(0.45–0.52)	-0.14(-0.14–-0.13)
Micronesia (Federated States of)	8(5–12)	19.87(11.32–28.43)		8(4–12)	22.25(10.82–33.68)		7(6–9)	20.93(17.54–24.31)		0.35(0.34–0.36)	-0.34(-0.34–-0.34)
Mongolia	80(38–122)	10.14(4.83–15.46)		118(64–172)	12(6.48–17.53)		174(149–199)	17.32(14.82–19.83)		0.54(0.51–0.56)	2.05(2.03–2.06)
Nauru	1(0–1)	23.03(13.56–32.49)		1(1–1)	24.42(12.89–35.96)		1(1–1)	22.28(16.23–28.32)		0.18(0.15–0.21)	-0.5(-0.51–-0.5)
New Zealand	140(108–171)	17.31(13.38–21.24)		216(187–245)	22.12(19.16–25.09)		172(131–213)	18.79(14.3–23.28)		0.76(0.73–0.8)	-0.91(-0.91–-0.9)
Niue	0(0–0)	22.86(12.84–32.88)		0(0–0)	24.73(15.5–33.97)		0(0–0)	25.28(19.77–30.8)		0.24(0.19–0.3)	0.13(0.12–0.13)
Palau	1(0–1)	17.39(10.18–24.6)		1(1–1)	19.69(16.55–22.84)		1(1–1)	22.57(18.83–26.31)		0.38(0.36–0.4)	0.76(0.76–0.76)
Papua New Guinea	213(103–323)	14.94(7.24–22.64)		686(307–1065)	20.92(9.36–32.49)		914(841–986)	23.26(21.41–25.1)		1.06(1.05–1.08)	0.59(0.59–0.59)
Philippines	613(388–838)	2.65(1.68–3.63)		3111(2166–4057)	9.06(6.3–11.81)		4602(3780–5424)	16.5(13.56–19.45)		3.91(3.88–3.93)	3.35(3.31–3.39)
Republic of Korea	1149(774–1525)	9.13(6.15–12.12)		1350(1153–1547)	19.96(17.05–22.87)		800(557–1042)	22.12(15.41–28.82)		2.45(2.38–2.52)	0.57(0.57–0.57)
Samoa	13(9–18)	20.42(13.64–27.19)		20(10–29)	25.99(13.52–38.46)		20(17–24)	25.61(20.93–30.29)		0.75(0.71–0.79)	-0.08(-0.08–-0.08)

Singapore	61(29–93)	9.07(4.24–13.9)		164(80–248)	19.56(9.59–29.54)		164(138–190)	22.59(18.96–26.23)		2.43(2.4–2.46)	0.8(0.8–0.8)
Solomon Islands	15(7–22)	11.16(5.55–16.77)		37(19–55)	13.67(7–20.33)		57(49–65)	15.67(13.48–17.86)		0.62(0.61–0.64)	0.76(0.76–0.77)
Taiwan	633(436–831)	10.85(7.47–14.24)		464(328–600)	14.98(10.59–19.37)		275(193–356)	15.23(10.7–19.77)		0.98(0.9–1.05)	0.1(0.1–0.1)
Tokelau	0(0–0)	22.36(12.7–32.01)		0(0–0)	23.75(11.77–35.73)		0(0–0)	21.26(13.38–29.14)		0.2(0.11–0.28)	-0.61(-0.61--0.61)
Tonga	9(6–13)	25.25(16.32–34.17)		9(6–12)	24(16.67–31.32)		5(3–7)	16.88(10.54–23.21)		-0.16(-0.19--0.12)	-1.94(-1.95--1.93)
Tuvalu	0(0–1)	20.27(10.89–29.65)		1(0–1)	22.1(11.87–32.33)		1(0–1)	19.31(15.26–23.35)		0.28(0.26–0.3)	-0.75(-0.75--0.75)
Vanuatu	7(3–10)	12.39(5.57–19.21)		19(8–31)	17.87(7.07–28.68)		31(26–37)	21.6(17.84–25.36)		1.16(1.12–1.2)	1.06(1.05–1.06)
Viet Nam	174(93–254)	0.74(0.4–1.09)		2832(2021–3644)	12.06(8.6–15.51)		4774(3645–5902)	23.83(18.2–29.46)		9.14(9.03–9.25)	3.79(3.74–3.84)

Note: Case numbers were rounded to the nearest thousand; therefore, a value of 0 may represent fewer than 500 cases rather than the absence of cases.

Supplementary Table S2. Prevalence and number of cases of obesity, and average annual percentage changes (AAPCs) in obesity prevalence, among children and adolescents aged 5 to 19 years, by WHO region and country or territory, from 1990 to 2040

Characteristics	1990		2022		2040		1990-2022	2023-2040
	Number of cases, thousands (95% CI)	Prevalence, % (95% CI)	Number of cases, thousands (95% CI)	Prevalence, % (95% CI)	Number of cases, thousands (95% CI)	Prevalence, % (95% CI)	AAPC, % (95% CI)	AAPC, % (95% CI)
**Africa**								
Algeria	447(86–808)	4.6(0.89–8.31)	1902(658–3146)	15.28(5.28–25.28)	3143(2717–3569)	23.97(20.72–27.21)	3.82(3.81–3.84)	2.51(2.48–2.53)
Angola	25(2–48)	0.6(0.04–1.16)	604(65–1142)	4.45(0.48–8.42)	2135(1678–2593)	9.32(7.32–11.32)	6.48(6.46–6.5)	4.12(4.07–4.18)
Benin	16(2–30)	0.79(0.08–1.49)	277(55–500)	5.45(1.07–9.82)	773(602–943)	10.43(8.13–12.73)	6.23(6.18–6.29)	3.63(3.59–3.68)
Botswana	4(0–8)	0.82(0.07–1.57)	45(8–83)	6.05(1.03–11.07)	113(89–136)	12.36(9.79–14.94)	6.45(6.4–6.49)	4(3.94–4.05)
Burkina Faso	5(1–10)	0.16(0.02–0.3)	94(22–167)	1.07(0.25–1.89)	278(206–350)	2.42(1.8–3.05)	6.19(6.11–6.27)	4.55(4.49–4.62)
Burundi	9(0–18)	0.42(0.02–0.82)	132(11–252)	2.42(0.2–4.65)	357(290–424)	4.7(3.82–5.59)	5.64(5.56–5.73)	3.71(3.66–3.76)
Cabo Verde	1(0–2)	0.71(0.05–1.37)	6(1–11)	4.19(0.76–7.62)	8(6–10)	8.41(6.6–10.23)	5.71(5.67–5.74)	3.9(3.85–3.95)
Cameroon	28(4–53)	0.65(0.09–1.22)	434(101–767)	4.24(0.99–7.49)	1420(1092–1748)	9.29(7.15–11.44)	6.03(5.99–6.06)	4.38(4.32–4.44)
Central African Republic	7(0–14)	0.68(0.04–1.33)	112(8–216)	5.05(0.35–9.76)	396(312–481)	10.46(8.23–12.68)	6.47(6.42–6.51)	4.06(4–4.11)
Chad	12(1–24)	0.53(0.03–1.04)	255(25–485)	3.49(0.34–6.63)	962(741–1183)	7.38(5.69–9.08)	6.07(5.99–6.14)	4.18(4.13–4.24)
Comoros	1(0–2)	0.73(0.06–1.39)	13(2–23)	4.42(0.66–8.18)	33(27–40)	8.8(7.04–10.56)	5.8(5.75–5.84)	3.85(3.8–3.9)
Congo	2(1–4)	0.23(0.06–0.41)	62(6–119)	2.8(0.26–5.34)	212(161–263)	6.7(5.09–8.31)	8.05(8.02–8.09)	4.87(4.79–4.94)
Cote d'Ivoire	38(4–72)	0.79(0.08–1.5)	608(132–1084)	5.42(1.17–9.66)	1665(1309–2021)	10.43(8.2–12.66)	6.21(6.12–6.29)	3.66(3.61–3.7)
Democratic Republic of the Congo	88(10–165)	0.67(0.08–1.26)	1806(262–3349)	4.62(0.67–8.58)	6685(5261–8109)	9.44(7.43–11.45)	6.24(6.2–6.28)	3.98(3.93–4.04)
Equatorial Guinea	1(0–2)	0.69(0.04–1.34)	30(2–58)	5.03(0.37–9.7)	91(72–109)	10.13(8.1–12.16)	6.41(6.39–6.43)	3.91(3.85–3.96)
Eritrea	4(0–7)	0.5(0.03–0.97)	43(4–83)	3.36(0.28–6.44)	115(91–139)	6.98(5.51–8.44)	6.13(6.11–6.16)	4.09(4.03–4.14)
Eswatini	4(1–8)	1.15(0.15–2.15)	27(6–48)	6.79(1.58–12.01)	53(43–63)	12.64(10.22–15.06)	5.71(5.69–5.73)	3.47(3.43–3.51)
Ethiopia	56(7–104)	0.32(0.04–0.6)	776(222–1331)	1.71(0.49–2.94)	2608(1981–3235)	3.86(2.93–4.79)	5.42(5.36–5.48)	4.53(4.47–4.6)
Gabon	2(0–5)	0.69(0.07–1.3)	42(10–75)	5.38(1.26–9.49)	126(99–152)	11.32(8.92–13.72)	6.65(6.63–6.67)	4.15(4.09–4.21)
Gambia	3(0–5)	0.69(0.05–1.33)	43(8–78)	4.36(0.85–7.86)	117(92–143)	8.83(6.9–10.76)	5.92(5.87–5.96)	3.94(3.89–3.99)
Ghana	74(15–133)	1.25(0.25–2.24)	792(482–1103)	7.02(4.27–9.77)	1945(1532–2359)	14.09(11.09–17.08)	5.57(5.55–5.6)	3.88(3.83–3.93)
Guinea	11(1–21)	0.47(0.04–0.91)	186(33–338)	3.57(0.63–6.52)	604(461–747)	8.26(6.3–10.21)	6.54(6.5–6.59)	4.66(4.59–4.74)

Guinea-Bissau	2(0–4)	0.61(0.04–1.17)		32(4–60)	4.14(0.47–7.82)		89(68–109)	8.82(6.79–10.84)		6.19(6.15–6.22)	4.21(4.15–4.27)
Kenya	56(5–107)	0.59(0.06–1.13)		490(176–803)	2.47(0.89–4.05)		687(419–955)	2.86(1.75–3.98)		4.55(4.51–4.59)	0.84(0.83–0.84)
Lesotho	6(1–12)	0.96(0.14–1.78)		36(8–63)	4.61(1.02–8.21)		66(53–78)	8.17(6.66–9.69)		5.02(4.99–5.05)	3.2(3.16–3.24)
Liberia	3(0–6)	0.39(0.03–0.74)		183(69–296)	9.09(3.45–14.73)		841(601–1082)	31.19(22.28–40.1)		10.45(10.17–10.73)	6.79(6.66–6.92)
Madagascar	14(0–27)	0.29(0.01–0.57)		240(19–461)	2.21(0.17–4.24)		777(595–958)	4.96(3.8–6.12)		6.56(6.49–6.62)	4.52(4.45–4.59)
Malawi	12(1–23)	0.34(0.04–0.65)		160(32–287)	1.99(0.39–3.59)		443(347–539)	4.05(3.17–4.92)		5.65(5.61–5.68)	3.95(3.9–4.01)
Mali	28(3–53)	0.79(0.09–1.49)		511(113–910)	5.51(1.21–9.8)		1619(1261–1977)	10.7(8.33–13.06)		6.25(6.19–6.32)	3.71(3.67–3.76)
Mauritania	9(1–17)	1.22(0.14–2.29)		149(49–249)	8.07(2.67–13.46)		439(351–527)	15.29(12.21–18.36)		6.09(6.05–6.14)	3.57(3.53–3.62)
Mauritius	5(1–9)	1.43(0.19–2.68)		28(15–42)	12.37(6.4–18.34)		37(30–43)	22.78(18.55–27.01)		6.98(6.95–7.02)	3.41(3.37–3.45)
Mozambique	24(4–45)	0.48(0.07–0.89)		212(43–380)	1.67(0.34–3)		596(456–735)	2.92(2.24–3.6)		3.93(3.86–3.99)	3.12(3.09–3.16)
Namibia	3(0–6)	0.62(0.07–1.17)		44(5–82)	4.66(0.57–8.75)		120(94–146)	9.82(7.73–11.91)		6.53(6.47–6.59)	4.15(4.1–4.21)
Niger	14(1–26)	0.44(0.03–0.85)		378(77–679)	3.69(0.75–6.62)		1545(1172–1918)	8.7(6.6–10.8)		6.89(6.82–6.95)	4.78(4.7–4.85)
Nigeria	197(33–360)	0.54(0.09–0.98)		3196(973–5420)	3.77(1.15–6.39)		9176(6972–11379)	8.07(6.14–10.01)		6.28(6.23–6.33)	4.25(4.19–4.31)
Rwanda	9(1–17)	0.3(0.03–0.58)		79(17–141)	1.61(0.34–2.87)		214(167–261)	3.33(2.59–4.06)		5.35(5.3–5.41)	4.07(4.01–4.12)
Sao Tome and Principe	0(0–1)	0.98(0.09–1.86)		3(1–6)	3.99(0.61–7.37)		6(4–8)	5.84(3.89–7.79)		4.48(4.43–4.54)	2.14(2.12–2.15)
Senegal	12(1–22)	0.38(0.03–0.73)		161(17–305)	2.5(0.27–4.73)		504(380–628)	5.85(4.41–7.3)		6.03(5.97–6.1)	4.74(4.67–4.81)
Seychelles	1(0–1)	2.41(0.89–3.92)		4(4–5)	18.07(14.81–21.32)		10(7–12)	34.6(26.6–42.61)		6.47(6.39–6.56)	3.62(3.57–3.66)
Sierra Leone	13(2–25)	0.89(0.11–1.68)		75(20–130)	2.52(0.67–4.36)		136(78–194)	3.49(2–4.99)		3.26(3.17–3.35)	1.84(1.83–1.85)
South Africa	83(26–140)	0.61(0.19–1.03)		1113(661–1566)	7.09(4.21–9.98)		1237(582–1894)	6.87(3.23–10.52)		7.99(7.84–8.14)	-0.17(-0.18–-0.16)
South Sudan	13(1–26)	0.72(0.04–1.4)		239(28–451)	5.39(0.62–10.15)		587(470–703)	10.8(8.65–12.95)		6.49(6.46–6.52)	3.89(3.84–3.94)
Togo	10(1–19)	0.65(0.05–1.25)		98(17–179)	2.99(0.51–5.48)		221(171–271)	4.86(3.75–5.96)		4.88(4.81–4.95)	2.71(2.69–2.74)
Uganda	23(3–43)	0.33(0.04–0.62)		869(300–1439)	4.64(1.6–7.67)		4617(3228–6005)	16.54(11.56–21.51)		8.61(8.54–8.68)	7(6.86–7.14)
United Republic of Tanzania	26(2–49)	0.25(0.02–0.48)		426(81–772)	1.75(0.33–3.18)		1564(1196–1931)	4.01(3.07–4.96)		6.27(6.22–6.32)	4.61(4.55–4.68)
Zambia	15(1–29)	0.48(0.04–0.92)		398(92–703)	5.17(1.2–9.14)		1384(1061–1706)	12.28(9.42–15.14)		7.72(7.69–7.75)	4.82(4.74–4.89)
Zimbabwe	23(3–44)	0.55(0.07–1.03)		152(29–275)	2.45(0.47–4.43)		345(262–428)	4.41(3.35–5.46)		4.77(4.69–4.85)	3.28(3.24–3.32)
**Americas**											
Antigua and Barbuda	1(0–2)	7.21(1.73–12.69)		4(2–7)	24.41(9.23–39.58)		5(5–6)	34.49(32.5–36.48)		3.88(3.84–3.92)	1.91(1.9–1.92)
Argentina	315(92–539)	3.36(0.98–5.74)		2208(1606–2811)	20.31(14.77–25.85)		3055(2448–3661)	40.13(32.16–48.09)		5.79(5.76–5.81)	3.79(3.74–3.84)

Bahamas	6(2–11)	7.7(2–13.4)		22(12–31)	26.83(14.9–38.76)		25(22–28)	37.75(33.71–41.8)		3.98(3.92–4.04)	1.88(1.87–1.9)
Barbados	4(1–7)	6.15(1.49–10.82)		12(5–19)	22.98(9.52–36.44)		15(14–16)	33.78(30.98–36.58)		4.2(4.17–4.23)	2.14(2.12–2.16)
Belize	4(1–8)	6.12(1.35–10.9)		25(9–40)	21.76(8.1–35.42)		35(32–38)	32.47(29.7–35.24)		4.04(4–4.07)	2.24(2.22–2.25)
Bermuda	1(0–1)	4.98(0.73–9.23)		2(0–3)	18.99(4.53–33.46)		2(2–2)	28.76(25.93–31.58)		4.27(4.24–4.3)	2.3(2.29–2.32)
Bolivia (Plurinational State of)	91(20–163)	3.61(0.79–6.43)		586(252–921)	16.33(7–25.65)		1148(955–1341)	29.36(24.42–34.3)		4.84(4.81–4.86)	3.27(3.23–3.31)
Brazil	1581(1107–2056)	3.17(2.22–4.12)		6839(5489–8189)	15.49(12.44–18.55)		8998(7445–10552)	24.53(20.3–28.76)		5.09(5.04–5.14)	2.56(2.54–2.58)
Canada	257(155–360)	4.46(2.68–6.24)		683(451–916)	10.95(7.22–14.69)		634(300–968)	10.09(4.78–15.4)		2.84(2.8–2.88)	-0.45(-0.46–-0.45)
Chile	257(107–407)	6.69(2.79–10.58)		1007(642–1372)	27.48(17.52–37.45)		1029(959–1099)	38.44(35.83–41.05)		4.51(4.45–4.57)	1.85(1.83–1.86)
Colombia	175(56–293)	1.6(0.51–2.68)		1200(754–1646)	10.54(6.62–14.45)		2224(1769–2678)	21.35(16.98–25.71)		6.11(6.05–6.18)	3.94(3.88–3.99)
Costa Rica	40(10–69)	3.99(1.04–6.93)		147(77–217)	13.77(7.19–20.35)		152(136–167)	20.08(18.03–22.12)		3.95(3.91–3.98)	2.1(2.09–2.12)
Cuba	137(51–223)	5.22(1.93–8.5)		310(204–417)	17.08(11.22–22.93)		340(258–423)	26.38(19.98–32.77)		3.78(3.68–3.88)	2.42(2.4–2.44)
Dominica	1(0–2)	5(1.2–8.79)		2(1–4)	18.69(6.35–31.02)		3(3–3)	29.5(25.8–33.21)		4.2(4.18–4.23)	2.54(2.52–2.56)
Dominican Republic	83(21–145)	3.19(0.8–5.59)		467(185–749)	15.49(6.13–24.85)		813(676–951)	28.1(23.35–32.85)		5.06(5.05–5.08)	3.32(3.28–3.36)
Ecuador	141(38–245)	3.77(1.01–6.53)		591(377–805)	12.61(8.05–17.18)		807(709–906)	19.89(17.46–22.32)		3.84(3.81–3.88)	2.54(2.51–2.56)
El Salvador	90(25–154)	4.44(1.25–7.62)		195(90–301)	11.63(5.35–17.91)		230(182–277)	16.59(13.17–20)		3.05(2.99–3.1)	1.97(1.96–1.99)
Grenada	2(0–3)	5.01(0.73–9.29)		5(1–8)	18.95(4.55–33.36)		6(5–6)	29.24(25.66–32.81)		4.24(4.21–4.27)	2.4(2.38–2.42)
Guatemala	93(28–158)	2.67(0.79–4.55)		449(319–579)	7.72(5.48–9.95)		617(559–676)	10.55(9.55–11.55)		3.36(3.34–3.38)	1.75(1.73–1.76)
Guyana	8(2–14)	2.89(0.57–5.21)		32(10–53)	13.72(4.55–22.9)		60(48–71)	25.4(20.62–30.18)		4.99(4.96–5.01)	3.43(3.39–3.47)
Haiti	85(13–157)	3.42(0.53–6.32)		408(96–720)	11.39(2.67–20.1)		638(584–693)	17.02(15.56–18.49)		3.83(3.79–3.87)	2.24(2.23–2.26)
Honduras	70(12–128)	3.56(0.63–6.5)		406(102–710)	12.62(3.16–22.07)		710(627–793)	19.78(17.46–22.1)		4.02(4–4.04)	2.51(2.49–2.53)
Jamaica	26(7–45)	3.26(0.91–5.6)		96(40–152)	15.5(6.5–24.5)		112(95–129)	26.68(22.65–30.71)		5(4.98–5.02)	3.03(3–3.06)
Mexico	1341(697–1985)	4.36(2.27–6.45)		5905(5025–6784)	17.7(15.06–20.33)		7335(6107–8564)	24.33(20.25–28.4)		4.46(4.42–4.51)	1.77(1.76–1.78)
Nicaragua	65(14–116)	3.81(0.84–6.78)		252(61–443)	12.74(3.07–22.41)		385(341–429)	19.41(17.18–21.63)		3.85(3.8–3.9)	2.34(2.33–2.36)
Panama	31(10–52)	3.7(1.25–6.16)		161(105–217)	14.28(9.3–19.26)		250(222–278)	22.6(20.06–25.13)		4.31(4.25–4.38)	2.56(2.54–2.58)
Paraguay	50(9–91)	3.46(0.6–6.31)		270(129–410)	14.55(6.97–22.12)		500(425–575)	24.96(21.22–28.7)		4.59(4.56–4.62)	3.01(2.98–3.04)
Peru	221(65–378)	2.78(0.81–4.75)		1427(1189–1665)	16.87(14.06–19.69)		2984(2317–3651)	36.87(28.63–45.12)		5.82(5.77–5.88)	4.35(4.29–4.41)
Puerto Rico	57(10–103)	5.81(1.03–10.59)		105(32–177)	21.27(6.6–35.94)		82(75–90)	31.82(28.79–34.85)		4.13(4.11–4.15)	2.24(2.22–2.26)
Saint Kitts and Nevis	1(0–1)	6.3(1.51–11.08)		2(1–3)	22.93(9.19–36.66)		3(2–3)	34.14(31.1–37.19)		4.11(4.09–4.14)	2.22(2.21–2.24)

Saint Lucia	3(0–5)	5.18(0.87–9.49)		7(3–11)	19.24(7.53–30.95)		8(7–9)	30.02(26.61–33.43)		4.18(4.16–4.21)	2.48(2.46–2.5)
Saint Vincent and the Grenadines	2(0–4)	5.41(1.16–9.67)		4(2–6)	18.54(9.9–27.19)		4(4–5)	26.82(24.59–29.05)		3.91(3.88–3.95)	2.06(2.04–2.07)
Suriname	7(1–13)	4.83(0.71–8.95)		30(8–52)	18.2(4.7–31.71)		46(40–52)	28.28(24.79–31.78)		4.24(4.16–4.31)	2.46(2.44–2.48)
Trinidad and Tobago	13(5–22)	3.35(1.18–5.52)		55(21–90)	19.7(7.35–32.05)		72(61–83)	32.34(27.47–37.22)		5.7(5.68–5.72)	2.76(2.73–2.78)
United States of America	6073(4999–7148)	11.52(9.48–13.56)		13338(10015–16662)	20.61(15.48–25.75)		12223(7514–16933)	20.7(12.73–28.68)		1.82(1.77–1.87)	0.03(0.02–0.03)
Uruguay	25(8–43)	3.22(0.98–5.45)		125(86–163)	17.77(12.3–23.23)		158(132–184)	32.09(26.79–37.39)		5.5(5.47–5.52)	3.3(3.26–3.33)
Venezuela (Bolivarian Republic of)	243(49–438)	3.53(0.72–6.35)		1198(439–1957)	14.91(5.46–24.36)		1616(1401–1832)	24.71(21.42–28)		4.6(4.58–4.62)	2.82(2.79–2.85)
**Europe**											
Albania	25(8–43)	2.51(0.76–4.27)		40(22–58)	7.74(4.23–11.26)		43(34–52)	13.07(10.39–15.75)		3.58(3.53–3.64)	2.91(2.88–2.94)
Andorra	1(0–1)	5.82(1.13–10.51)		1(0–2)	11.14(1.91–20.38)		1(1–1)	11.5(7.55–15.44)		2.05(1.96–2.14)	0.17(0.17–0.18)
Armenia	44(11–78)	4.62(1.1–8.14)		53(28–79)	9.37(4.87–13.86)		65(47–83)	15.36(11.15–19.57)		2.23(2.13–2.33)	2.76(2.73–2.78)
Austria	51(33–68)	3.58(2.36–4.8)		138(102–174)	10.55(7.8–13.29)		142(109–175)	12.5(9.57–15.43)		3.42(3.38–3.46)	0.94(0.93–0.94)
Azerbaijan	68(10–126)	3.12(0.47–5.77)		202(46–358)	8.55(1.94–15.16)		303(231–376)	16.08(12.23–19.92)		3.19(3.13–3.26)	3.52(3.47–3.56)
Belarus	66(9–124)	2.92(0.38–5.46)		127(29–225)	8.07(1.81–14.33)		98(71–125)	10.78(7.84–13.71)		3.24(3.15–3.33)	1.6(1.59–1.61)
Belgium	70(27–114)	3.77(1.44–6.1)		130(70–189)	6.53(3.54–9.52)		111(84–139)	6.93(5.23–8.63)		1.73(1.65–1.81)	0.33(0.32–0.33)
Bosnia and Herzegovina	42(9–74)	3.76(0.83–6.7)		54(20–88)	11.68(4.35–19.01)		62(45–78)	18.49(13.62–23.35)		3.62(3.48–3.77)	2.56(2.53–2.58)
Bulgaria	75(51–99)	3.98(2.71–5.25)		122(85–159)	12.12(8.43–15.81)		139(102–175)	16.47(12.14–20.8)		3.56(3.5–3.62)	1.7(1.69–1.71)
Croatia	41(16–66)	4.16(1.65–6.67)		54(34–74)	9.23(5.87–12.59)		40(19–61)	8.91(4.27–13.56)		2.53(2.47–2.58)	-0.2(-0.21–-0.2)
Cyprus	13(5–21)	6.79(2.54–11.04)		29(18–40)	14.1(8.74–19.46)		35(28–43)	16.19(12.72–19.66)		2.31(2.27–2.36)	0.77(0.76–0.77)
Czechia	59(24–94)	2.47(1.02–3.92)		99(60–139)	5.94(3.56–8.33)		77(39–115)	5.9(3.01–8.8)		2.81(2.73–2.89)	-0.04(-0.05–-0.04)
Denmark	45(33–57)	4.74(3.48–6)		55(34–75)	5.56(3.46–7.65)		62(35–89)	6.67(3.71–9.62)		0.48(0.41–0.55)	1.02(1.01–1.02)
Estonia	8(2–14)	2.37(0.61–4.14)		23(17–30)	10.67(7.87–13.48)		24(17–31)	16.16(11.39–20.93)		4.86(4.76–4.95)	2.3(2.29–2.32)
Finland	29(17–42)	3.06(1.78–4.35)		116(82–149)	12.68(9.04–16.33)		165(133–196)	23.99(19.35–28.62)		4.54(4.47–4.62)	3.55(3.51–3.6)
France	411(157–666)	3.43(1.31–5.55)		493(235–751)	4.12(1.96–6.28)		302(68–535)	2.98(0.67–5.29)		0.55(0.49–0.62)	-1.79(-1.81–-1.78)
Georgia	52(9–95)	3.99(0.69–7.29)		81(43–120)	10.61(5.59–15.63)		132(103–160)	20.66(16.18–25.13)		3.09(3–3.17)	3.71(3.66–3.75)
Germany	603(338–869)	4.68(2.62–6.74)		979(530–1428)	8.47(4.58–12.35)		952(646–1259)	9.02(6.12–11.92)		1.85(1.79–1.91)	0.35(0.35–0.36)

Greece	127(72–183)	5.74(3.24–8.25)		180(127–233)	11.62(8.19–15.04)		84(5–162)	7.98(0.49–15.48)		2.24(2.2–2.29)	-2.07(-2.08–-2.05)
Greenland	0(0–1)	3.63(0.72–6.54)		1(0–1)	4.74(0.91–8.57)		0(0–1)	4.39(2.39–6.39)		0.82(0.77–0.87)	-0.42(-0.43–-0.42)
Hungary	81(27–135)	3.55(1.17–5.93)		213(161–265)	14.82(11.19–18.44)		298(249–347)	23.52(19.66–27.38)		4.58(4.54–4.62)	2.58(2.55–2.6)
Iceland	3(0–5)	4.08(0.67–7.48)		6(1–11)	8.67(1.22–16.12)		8(7–10)	11.74(9.63–13.85)		2.39(2.34–2.44)	1.69(1.68–1.7)
Ireland	47(18–76)	4.67(1.81–7.53)		86(57–114)	8.31(5.57–11.05)		73(43–103)	8.27(4.84–11.7)		1.78(1.72–1.85)	-0.03(-0.03–-0.02)
Israel	50(34–66)	3.69(2.52–4.85)		220(138–302)	9.27(5.82–12.73)		319(225–412)	11.25(7.95–14.55)		2.93(2.9–2.95)	1.08(1.07–1.08)
Italy	825(571–1078)	7.52(5.2–9.83)		784(499–1069)	9.62(6.12–13.11)		320(50–589)	5.54(0.87–10.21)		0.79(0.75–0.84)	-3.03(-3.07–-2.99)
Kazakhstan	197(61–333)	3.95(1.22–6.69)		296(171–421)	5.66(3.27–8.05)		475(339–611)	7.6(5.43–9.78)		1.13(1.03–1.24)	1.64(1.63–1.65)
Kyrgyzstan	116(68–164)	7.99(4.69–11.29)		44(14–75)	2.17(0.69–3.65)		15(8–21)	0.62(0.35–0.90)		-4.01(-4.07–-3.95)	-6.58(-6.81–-6.35)
Latvia	14(3–24)	2.49(0.63–4.34)		20(11–30)	6.7(3.61–9.8)		18(10–25)	8.82(5.06–12.59)		3.13(3.01–3.24)	1.53(1.53–1.54)
Lithuania	23(6–40)	2.78(0.72–4.83)		30(16–44)	7.06(3.76–10.37)		25(19–31)	8.53(6.47–10.59)		2.95(2.89–3.01)	1.03(1.02–1.04)
Luxembourg	2(0–4)	3.32(0.53–6.11)		8(1–14)	7.44(1–13.88)		13(10–15)	11.16(9.24–13.08)		2.55(2.5–2.6)	2.26(2.24–2.28)
Malta	6(2–10)	6.89(2.44–11.34)		8(5–12)	12.05(6.94–17.17)		7(5–9)	11.1(7.66–14.54)		1.77(1.73–1.81)	-0.46(-0.46–-0.45)
Montenegro	6(3–10)	4.07(2.09–6.06)		10(7–13)	8.73(6.47–10.98)		10(8–13)	11.26(8.3–14.21)		2.42(2.34–2.49)	1.41(1.41–1.42)
Netherlands (Kingdom of the)	74(25–123)	2.57(0.87–4.27)		137(50–224)	4.75(1.74–7.76)		191(152–230)	6.81(5.43–8.19)		1.91(1.85–1.97)	2.01(1.99–2.02)
North Macedonia	22(7–38)	4.04(1.32–6.76)		37(21–52)	11.45(6.58–16.31)		36(27–45)	16.05(12.2–19.9)		3.32(3.29–3.35)	1.88(1.86–1.89)
Norway	27(11–42)	3.17(1.35–5)		59(31–87)	6.18(3.27–9.09)		63(45–82)	7.69(5.47–9.91)		2.08(2.02–2.14)	1.22(1.22–1.23)
Poland	270(172–367)	2.85(1.82–3.88)		429(283–575)	7.26(4.78–9.73)		300(186–414)	7.33(4.55–10.11)		2.97(2.93–3.01)	0.05(0.04–0.05)
Portugal	106(70–142)	4.59(3.02–6.15)		110(69–151)	7.57(4.76–10.38)		61(37–86)	4.67(2.79–6.55)		1.55(1.41–1.69)	-2.65(-2.68–-2.63)
Republic of Moldova	20(4–36)	1.78(0.36–3.19)		28(8–49)	4.76(1.33–8.19)		31(24–37)	7.57(5.97–9.16)		3.13(3.06–3.21)	2.58(2.56–2.6)
Romania	59(23–95)	1.08(0.42–1.73)		278(200–357)	8.83(6.33–11.32)		357(242–472)	14.06(9.53–18.59)		6.86(6.64–7.08)	2.59(2.56–2.61)
Russian Federation	1321(895–1746)	4.03(2.73–5.32)		1610(998–2221)	6.25(3.88–8.63)		631(327–936)	3.28(1.70–4.86)		1.42(1.26–1.59)	-3.53(-3.58–-3.49)
Serbia	70(41–99)	4.45(2.61–6.3)		108(71–145)	10.76(7.04–14.48)		116(95–137)	14.16(11.56–16.76)		2.78(2.75–2.81)	1.53(1.53–1.54)
Slovakia	34(18–50)	2.49(1.3–3.69)		84(49–118)	9.8(5.76–13.84)		82(52–112)	11.66(7.4–15.92)		4.39(4.33–4.46)	0.96(0.96–0.97)
Slovenia	18(16–20)	4.12(3.63–4.61)		35(30–39)	10.87(9.37–12.37)		33(22–44)	12.61(8.32–16.89)		3.08(3.05–3.11)	0.82(0.81–0.82)

Spain	603(332–875)	6.74(3.71–9.77)		752(543–960)	10.48(7.57–13.39)		434(270–599)	8.64(5.37–11.91)		1.4(1.36–1.44)	-1.07(-1.07–-1.06)
Sweden	60(49–71)	3.88(3.2–4.57)		187(120–254)	10.13(6.51–13.75)		273(209–338)	17.45(13.33–21.57)		3.05(2.99–3.11)	3.03(3–3.06)
Switzerland	44(18–70)	3.72(1.5–5.94)		91(67–116)	6.96(5.11–8.81)		97(77–116)	7.68(6.12–9.24)		1.98(1.92–2.04)	0.55(0.54–0.55)
Tajikistan	26(3–49)	1.37(0.18–2.57)		51(18–84)	1.58(0.57–2.59)		96(77–116)	2.35(1.88–2.83)		0.43(0.39–0.47)	2.23(2.22–2.25)
Turkey	500(135–865)	2.61(0.71–4.52)		2307(1255–3359)	11.73(6.38–17.08)		3252(2647–3857)	21.72(17.68–25.76)		4.81(4.76–4.85)	3.44(3.4–3.48)
Turkmenistan	30(4–56)	2.29(0.33–4.26)		67(20–115)	3.41(0.99–5.84)		135(108–161)	5.97(4.78–7.15)		1.25(1.21–1.3)	3.11(3.08–3.14)
Ukraine	175(41–309)	1.6(0.38–2.82)		286(174–399)	4.39(2.66–6.12)		168(54–283)	4.68(1.5–7.87)		3.22(3.08–3.36)	0.36(0.35–0.36)
United Kingdom	505(394–616)	4.5(3.51–5.49)		1386(1057–1716)	11.33(8.63–14.02)		1513(896–2130)	13.38(7.92–18.83)		2.91(2.83–2.99)	0.93(0.92–0.93)
Uzbekistan	157(24–290)	2.21(0.33–4.09)		587(102–1072)	6.44(1.12–11.75)		1803(1406–2199)	13.1(10.22–15.98)		3.4(3.33–3.47)	3.96(3.9–4.01)
**Eastern Mediterranean**											
Afghanistan	35(3–67)	0.76(0.07–1.45)		1333(444–2222)	8.47(2.82–14.13)		4654(3615–5693)	19.18(14.9–23.47)		7.84(7.81–7.87)	4.55(4.48–4.62)
Bahrain	9(3–14)	6.12(2.33–9.91)		57(33–82)	20.12(11.58–28.67)		93(81–106)	29.25(25.43–33.08)		3.8(3.77–3.82)	2.08(2.07–2.1)
Djibouti	1(0–2)	0.55(0.03–1.08)		12(1–23)	3.44(0.26–6.62)		26(20–32)	7.13(5.56–8.7)		5.88(5.85–5.91)	4.08(4.02–4.13)
Egypt	2034(957–3112)	9.82(4.62–15.03)		7202(4486–9918)	20.42(12.72–28.12)		10721(8948–12494)	27.9(23.29–32.52)		2.32(2.26–2.39)	1.73(1.72–1.74)
Iran (Islamic Republic of)	469(288–649)	2.18(1.34–3.03)		2566(1813–3318)	12.63(8.92–16.33)		3654(2958–4349)	22.03(17.84–26.23)		5.62(5.58–5.67)	3.1(3.07–3.14)
Iraq	379(90–669)	5.47(1.3–9.64)		2910(1242–4578)	18.66(7.96–29.36)		5542(4842–6241)	29.22(25.53–32.9)		3.92(3.87–3.97)	2.5(2.48–2.52)
Jordan	74(24–124)	5.18(1.66–8.7)		621(387–855)	17.79(11.09–24.48)		1017(855–1178)	27.47(23.11–31.83)		3.94(3.9–3.98)	2.42(2.4–2.44)
Kuwait	61(35–86)	10.92(6.34–15.51)		182(110–254)	21.11(12.77–29.45)		206(92–320)	21(9.41–32.6)		2(1.87–2.14)	-0.03(-0.04–-0.03)
Lebanon	54(22–85)	4.44(1.83–7.04)		300(116–485)	18.57(7.15–29.99)		412(353–471)	28.67(24.56–32.77)		4.59(4.53–4.65)	2.41(2.39–2.43)
Libya	77(20–133)	4.34(1.14–7.55)		318(95–540)	15.43(4.62–26.24)		487(423–550)	24.8(21.57–28.03)		4.04(4.02–4.06)	2.64(2.62–2.67)
Morocco	207(42–372)	2.27(0.46–4.08)		556(270–843)	5.73(2.78–8.67)		732(593–870)	7.93(6.43–9.43)		2.92(2.86–2.99)	1.82(1.81–1.83)
State of Palestine	28(7–49)	3.27(0.87–5.67)		253(128–378)	13.53(6.83–20.23)		566(464–668)	24.59(20.15–29.03)		4.54(4.51–4.56)	3.34(3.3–3.37)
Oman	29(7–51)	4.51(1.14–7.88)		202(101–302)	18.84(9.43–28.25)		484(405–563)	32.75(27.39–38.11)		4.57(4.55–4.58)	3.08(3.05–3.12)
Pakistan	217(99–335)	0.51(0.23–0.8)		7200(4103–10297)	8.49(4.84–12.14)		24359(18090–30629)	23.22(17.24–29.19)		9.19(9.09–9.29)	5.58(5.48–5.67)
Qatar	8(2–14)	7.52(2.24–12.81)		93(41–145)	24.05(10.6–37.5)		185(164–206)	33.44(29.6–37.29)		3.68(3.62–3.75)	1.85(1.83–1.86)
Saudi Arabia	153(100–206)	4.22(2.75–5.69)		1366(690–2041)	18.03(9.11–26.95)		2528(2049–3006)	27.7(22.46–32.95)		4.64(4.58–4.7)	2.4(2.38–2.42)
Somalia	15(1–29)	0.56(0.03–1.09)		251(18–484)	3.61(0.25–6.97)		947(728–1165)	7.65(5.88–9.42)		5.99(5.94–6.05)	4.19(4.13–4.25)
Sudan	62(4–120)	0.74(0.05–1.42)		1147(232–2062)	6.37(1.29–11.46)		3562(2794–4330)	13.8(10.82–16.78)		6.98(6.95–7.02)	4.31(4.25–4.37)

Syrian Arab Republic	243(38–449)	4.81(0.75–8.86)		1239(269–2209)	15.99(3.48–28.51)		2425(2140–2710)	24.45(21.58–27.33)		3.83(3.81–3.85)	2.36(2.34–2.38)
Tunisia	54(21–87)	1.84(0.71–2.97)		312(91–534)	10.78(3.13–18.43)		491(395–588)	21.22(17.06–25.37)		5.69(5.66–5.73)	3.77(3.73–3.82)
United Arab Emirates	40(14–66)	8.01(2.78–13.25)		350(201–498)	21.54(12.39–30.69)		591(488–693)	28.51(23.58–33.43)		3.12(3.06–3.17)	1.56(1.55–1.57)
Yemen	139(43–235)	2.48(0.76–4.2)		1485(457–2512)	10.88(3.35–18.41)		5008(3970–6046)	22.6(17.91–27.28)		4.73(4.71–4.76)	4.07(4.02–4.13)
**South-East Asia**											
Bangladesh	103(8–198)	0.25(0.02–0.47)		943(378–1507)	1.9(0.76–3.04)		2167(1665–2669)	4.29(3.3–5.29)		6.59(6.54–6.64)	4.54(4.48–4.61)
Bhutan	2(0–3)	0.76(0.05–1.47)		11(3–20)	5.87(1.45–10.3)		18(14–21)	11.98(9.62–14.35)		6.59(6.57–6.61)	3.98(3.93–4.04)
Democratic People's Republic of Korea	58(5–111)	0.99(0.08–1.91)		457(67–847)	9.17(1.35–16.99)		866(690–1042)	18.36(14.64–22.08)		7.18(7.14–7.23)	3.87(3.82–3.92)
India	374(190–558)	0.13(0.06–0.19)		12944(8488–17400)	3.43(2.25–4.62)		24366(18114–30618)	7.14(5.31–8.97)		10.89(10.63–11.15)	4.08(4.03–4.14)
Indonesia	564(262–867)	0.89(0.41–1.37)		7664(4638–10690)	10.83(6.56–15.11)		16902(12976–20828)	25.22(19.36–31.07)		8.14(8.1–8.19)	4.7(4.63–4.77)
Maldives	1(0–2)	1.27(0.19–2.35)		14(7–21)	13.14(6.51–19.77)		22(17–26)	27.42(21.42–33.41)		7.56(7.51–7.6)	4.09(4.03–4.14)
Myanmar	115(13–217)	0.84(0.1–1.59)		986(209–1762)	7.41(1.57–13.24)		1914(1526–2302)	14.93(11.9–17.96)		7.01(6.97–7.05)	3.91(3.86–3.96)
Nepal	30(3–57)	0.43(0.04–0.81)		168(34–303)	1.9(0.38–3.42)		257(197–316)	3.23(2.48–3.98)		4.78(4.73–4.83)	2.97(2.94–3)
Sri Lanka	35(5–66)	0.65(0.09–1.22)		409(153–664)	7.67(2.88–12.46)		791(625–958)	16.55(13.07–20.04)		8.03(7.99–8.06)	4.29(4.23–4.35)
Thailand	285(168–402)	1.67(0.98–2.35)		1499(1129–1868)	12.17(9.17–15.17)		1657(1415–1900)	19.21(16.4–22.03)		6.41(6.21–6.6)	2.54(2.52–2.56)
Timor-Leste	1(0–1)	0.23(0.02–0.44)		4(1–7)	0.87(0.17–1.57)		7(5–10)	1.5(0.95–2.06)		4.23(4.09–4.38)	3.04(3.01–3.07)
**Western Pacific**											
American Samoa	1(1–2)	9.2(3.7–14.7)		4(2–7)	29.22(11.64–46.8)		4(3–4)	44.24(38.3–50.17)		3.68(3.67–3.69)	2.31(2.29–2.32)
Australia	206(148–265)	5.27(3.78–6.77)		755(537–974)	15.77(11.21–20.32)		1194(973–1414)	24.26(19.78–28.74)		3.48(3.42–3.54)	2.4(2.38–2.42)
Brunei Darussalam	2(1–4)	3.14(0.74–5.53)		22(15–29)	22.08(14.92–29.24)		31(26–37)	34.22(28.06–40.39)		6.26(6.17–6.36)	2.45(2.43–2.47)
Cambodia	18(2–35)	0.73(0.07–1.39)		297(75–520)	5.96(1.5–10.43)		618(502–735)	11.48(9.32–13.63)		6.77(6.68–6.86)	3.66(3.62–3.71)
China	3108(2369–3847)	0.95(0.72–1.17)		30816(22459–39174)	11.92(8.69–15.15)		28323(21808–34838)	22.7(17.48–27.92)		8.25(8.03–8.47)	3.59(3.54–3.63)
Cook Islands	1(0–1)	10.66(3.5–17.82)		1(1–2)	37.52(24.15–50.9)		1(1–1)	56.48(48.74–64.22)		4.01(3.97–4.05)	2.28(2.26–2.3)
Fiji	7(1–12)	2.52(0.49–4.54)		31(13–48)	12.27(5.33–19.21)		55(45–64)	22.88(18.8–26.95)		5.08(5.06–5.1)	3.48(3.43–3.52)
French Polynesia	6(2–10)	8.7(2.48–14.92)		17(9–24)	26.61(14.74–38.48)		15(13–17)	36.7(31.88–41.52)		3.55(3.48–3.62)	1.79(1.78–1.8)
Japan	843(719–967)	3.22(2.75–3.7)		710(505–915)	4.37(3.11–5.64)		857(620–1094)	7.42(5.37–9.48)		0.93(0.85–1.01)	2.95(2.92–2.98)
Kiribati	1(0–2)	4.62(1.06–8.18)		8(3–13)	18.81(6.36–31.26)		16(13–18)	32.36(27.11–37.61)		4.49(4.48–4.5)	3.03(2.99–3.06)

Lao People's Democratic Republic	13(1–24)	0.78(0.07–1.5)		154(22–286)	6.81(0.97–12.65)		339(268–410)	14.18(11.21–17.15)		6.99(6.94–7.04)	4.09(4.03–4.14)
Malaysia	149(62–235)	2.46(1.02–3.89)		1396(982–1810)	16.5(11.61–21.4)		1924(1567–2281)	26.65(21.7–31.6)		6.09(6.01–6.17)	2.68(2.65–2.71)
Marshall Islands	1(0–1)	3.95(0.92–6.98)		3(1–4)	18.9(7.88–29.92)		3(2–3)	32.88(27.33–38.42)		5.02(5–5.04)	3.08(3.05–3.12)
Micronesia (Federated States of)	2(1–4)	5.29(1.25–9.32)		7(2–12)	20.46(6.76–34.17)		12(10–14)	33.84(29.19–38.5)		4.32(4.31–4.33)	2.81(2.78–2.84)
Mongolia	20(3–36)	2.47(0.35–4.59)		64(20–108)	6.49(2–10.97)		135(100–169)	13.45(10.01–16.89)		3.03(2.91–3.16)	4.05(4–4.11)
Nauru	0(0–1)	10.19(3.5–16.87)		1(1–2)	32.94(17.64–48.23)		2(2–2)	47.21(41.43–53)		3.75(3.71–3.8)	2(1.99–2.02)
New Zealand	38(21–55)	4.73(2.64–6.82)		173(142–205)	17.79(14.58–21)		202(149–255)	22.08(16.26–27.89)		4.22(4.13–4.31)	1.2(1.19–1.21)
Niue	0(0–0)	11.17(3.45–18.88)		0(0–0)	38.71(26.32–51.1)		0(0–0)	58.79(51.11–66.48)		3.96(3.89–4.04)	2.33(2.31–2.35)
Palau	0(0–1)	7.85(2.73–12.98)		1(1–1)	28.05(24.03–32.06)		1(1–1)	42.98(37.12–48.85)		4.06(4.01–4.11)	2.38(2.36–2.4)
Papua New Guinea	52(9–95)	3.64(0.62–6.66)		505(111–899)	15.39(3.38–27.4)		1005(868–1142)	25.57(22.09–29.06)		4.61(4.6–4.63)	2.84(2.81–2.87)
Philippines	129(47–210)	0.56(0.21–0.91)		2028(1205–2850)	5.9(3.51–8.3)		3814(2914–4715)	13.68(10.45–16.91)		7.64(7.59–7.69)	4.68(4.61–4.75)
Republic of Korea	304(145–463)	2.41(1.15–3.68)		946(768–1124)	13.99(11.36–16.62)		1019(638–1401)	28.18(17.63–38.73)		5.59(5.43–5.74)	3.9(3.85–3.94)
Samoa	4(1–6)	5.49(1.94–9.05)		18(7–28)	23.41(8.91–37.92)		29(26–33)	37.14(33.03–41.26)		4.63(4.59–4.67)	2.58(2.56–2.6)
Singapore	24(5–43)	3.59(0.78–6.4)		129(43–214)	15.33(5.08–25.58)		190(156–225)	26.21(21.45–30.97)		4.64(4.6–4.68)	2.99(2.96–3.02)
Solomon Islands	2(0–4)	1.53(0.22–2.84)		24(7–41)	8.85(2.62–15.08)		73(56–89)	20.18(15.62–24.75)		5.66(5.61–5.71)	4.59(4.52–4.66)
Taiwan	172(84–259)	2.94(1.44–4.45)		335(204–465)	10.8(6.59–15)		251(112–390)	13.92(6.2–21.64)		4.08(3.94–4.22)	1.42(1.42–1.43)
Tokelau	0(0–0)	10.27(3.08–17.47)		0(0–0)	32.53(15.82–49.23)		0(0–0)	45.38(40.79–49.96)		3.69(3.61–3.77)	1.84(1.83–1.86)
Tonga	3(1–5)	8.03(2.75–13.3)		12(8–16)	32.57(22.32–42.82)		16(14–19)	52.47(45.39–59.55)		4.48(4.46–4.51)	2.66(2.63–2.68)
Tuvalu	0(0–0)	8.02(2.14–13.9)		1(0–1)	29.59(15.41–43.77)		1(1–1)	44.68(39.19–50.16)		4.17(4.15–4.2)	2.29(2.27–2.31)
Vanuatu	1(0–3)	2.6(0.3–4.89)		13(2–25)	12.53(2.01–23.05)		33(27–38)	22.53(18.83–26.23)		5.04(5.03–5.06)	3.27(3.23–3.31)
Viet Nam	28(10–47)	0.12(0.04–0.2)		1515(881–2150)	6.45(3.75–9.15)		2669(1967–3371)	13.32(9.82–16.83)		13.32(13.16–13.49)	4.02(3.97–4.08)
